# Resolving the heterogeneity of diaphragmatic mesenchyme: a novel mouse model of congenital diaphragmatic hernia

**DOI:** 10.1242/dmm.046797

**Published:** 2021-01-26

**Authors:** Louise Cleal, Sophie L. McHaffie, Martin Lee, Nick Hastie, Ofelia M. Martínez-Estrada, You-Ying Chau

**Affiliations:** 1 MRC Human Genetics Unit, Institute of Genetics and Molecular Medicine, University of Edinburgh, Edinburgh EH4 2XU, UK; 2 Molecular Pathology, Department of Laboratory Medicine, Royal Infirmary of Edinburgh, 51 Little France Crescent, Old Dalkeith Road, Edinburgh EH16 4SA, UK; 3 Cancer Research UK Edinburgh Centre, Institute of Genetics and Molecular Medicine, University of Edinburgh, Edinburgh EH4 2XR, UK; 4 Department of Cell Biology, Physiology and Immunology, Faculty of Biology, University of Barcelona, Av. Diagonal, 643, 08028 Barcelona, Spain; 5 Institute of Biomedicine (IBUB), University of Barcelona, Barcelona 08028, Spain; 6 Centre for Cardiovascular Science, Queen's Medical Research Institute, University of Edinburgh, Edinburgh EH16 4TJ, UK

**Keywords:** Congenital diaphragmatic hernia, *Wt1*, Diaphragm development, *Prx1-Cre*, Mesenchyme, Mouse model

## Abstract

Congenital diaphragmatic hernia (CDH) is a relatively common developmental defect with considerable mortality and morbidity. Formation of the diaphragm is a complex process that involves several cell types, each with different developmental origins. Owing to this complexity, the aetiology of CDH is not well understood. The pleuroperitoneal folds (PPFs) and the posthepatic mesenchymal plate (PHMP) are transient structures that are essential during diaphragm development. Using several mouse models, including lineage tracing, we demonstrate the heterogeneous nature of the cells that make up the PPFs. The conditional deletion of Wilms tumor 1 homolog (*Wt1*) in the non-muscle mesenchyme of the PPFs results in CDH. We show that the fusion of the PPFs and the PHMP to form a continuous band of tissue involves movements of cells from both sources. The PPFs of mutant mice fail to fuse with the PHMP and exhibit increased RALDH2 (also known as ALDH1A2) expression. However, no changes in the expression of genes (including *Snai1*, *Snai2*, *Cdh1* and *Vim*) implicated in epithelial-to-mesenchymal transition are observed. Additionally, the mutant PPFs lack migrating myoblasts and muscle connective tissue fibroblasts (TCF4^+^/GATA4^+^), suggesting possible interactions between these cell types. Our study demonstrates the importance of the non-muscle mesenchyme in development of the diaphragm.

## Abstract (precis)

**Summary:** We manipulated the expression of the developmental regulator *Wt1* in mesenchymal cells in a tissue-specific manner using transgenic mouse models, showing that mutant embryos can survive *in utero* but die shortly after birth owing to diaphragmatic hernia.

## INTRODUCTION

Congenital diaphragmatic hernia (CDH) is a severe developmental defect that affects approximately one in 3000 live births (Greer, 2013) and can have devastating clinical outcomes. Despite advances in surgical repair and neonatal care, the mortality remains high. There is an urgent need to develop new therapies by gaining a better understanding of the pathophysiology of CDH (Reiss et al., 2010). The aetiology of CDH is not well understood owing to the complexity of diaphragm formation (Kardon et al., 2017). The mature diaphragm is composed of several tissues, including connective tissue, muscle progenitors, muscle, tendons, nerves, blood vessels, lymphatics and mesothelium (Ackerman and Greer, 2007). Several embryonic structures are implicated in diaphragm development: the pleuroperitoneal folds (PPFs), the septum transversum (ST), the posthepatic mesenchymal plate (PHMP) and the somites (Carmona et al., 2016; Merrell and Kardon, 2013; Sefton et al., 2018). The ST is a thin layer of mesodermal cells overlying the liver and is formed at approximately embryonic day (E) 8.5 in the mouse. This is followed by the formation of the PPFs, which develop at E10.5-E12.5. A study demonstrated that the fibroblasts of the PPFs play a crucial role in guiding the expansion and movement of the myogenic cells that originate in the somites and are essential for the correct formation of the muscular component of the diaphragm (Merrell et al., 2015). The PHMP first appears at ∼E10.5 and is thought to be derived from the mesenchymal population of the lateral ST (Carmona et al., 2016; Iritani, 1984). The cells of PPFs fuse with the PHMP to form a membranous continuum, separating the thoracic and peritoneal cavities. Subsequently, myoblasts migrate from the somites, leading to the muscularisation of this membrane to form the mature diaphragm.

Several genes have been shown to be involved in normal diaphragm development and are associated with human CDH (Kardon et al., 2017). One of these is WT1 transcription factor (*WT1*). Mutations in *WT1* have been described in patients with CDH (Antonius et al., 2008; Holder et al., 2007; Scott et al., 2005; Suri et al., 2007). In the mouse, Wilms tumor 1 homolog (WT1) was first described as a transcriptional regulator with a large array of target genes, but it also has RNA-editing functions (Bharathavikru et al., 2017; Hastie, 2017; Toska and Roberts, 2014). *Wt1* plays an essential role in the development of multiple organs (Kreidberg et al., 1993; Moore et al., 1999) and is also crucial for maintaining adult tissue homeostasis (Chau et al., 2011). Homozygous *Wt1* null mouse embryos die at ∼E13.5 and have diaphragmatic hernias (Clugston and Greer, 2007; Kreidberg et al., 1993). During diaphragm development in the mouse, *Wt1* is expressed in the PPFs, PHMP, ST, mesothelium and lateral wall body mesenchyme (Carmona et al., 2016; Paris et al., 2016). Given the wide expression of *Wt1* in structures that are involved in diaphragm development, a tissue-specific approach is essential for delineating the role of *Wt1* and the role of the cells that express *Wt1* in the underlying pathophysiology of CDH.

Mesenchymal cells are present throughout the diaphragm, but their origins and cell types are not well defined or understood. One mesenchymal cell population, the connective tissue fibroblasts, for which GATA binding protein 4 (GATA4) and transcription factor 4 (TCF4) are the best markers (Merrell et al., 2015; Paris et al., 2016), is crucial for guiding the migration of myoblasts during diaphragm development, as shown by the conditional deletion of *Gata4* using the *Prx1-Cre* mouse model (Merrell et al., 2015). The TCF4/GATA4-expressing connective tissue fibroblast population does not overlap substantially with the WT1-expressing non-muscle mesenchyme in the diaphragm (Paris et al., 2016), suggesting that they are distinct cell populations. Furthermore, it has been shown previously that WT1^+^ mesenchymal cells largely corresponded to TWIST1, but that still explains only at most a 70% co-expression in an E13.5 diaphragm, providing further evidence of the complexity and heterogeneity of the mesenchymal cells that make up diaphragm (Paris et al., 2016).

To delineate the heterogeneity of the ill-defined mesenchymal cells in the diaphragm, we generated a mouse model in which *Wt1* was deleted conditionally in the *Prx1-Cre* lineage. In this model, mutant embryos can survive *in utero* but die shortly after birth, which we believe is attributable to the formation of diaphragmatic hernias. In addition to the CDH phenotype, we show that the developmental origin(s) of the non-muscle mesenchymal cells in the PPF is different from those in the PHMP. Moreover, we show data providing cellular insights into the roles of PPF mesenchymal cells during the formation of diaphragm.

## RESULTS

### Diaphragm development is disrupted in *Prx1^Cre^*^/+^*;Wt1^loxp/loxp^* embryos

In our model, male *Prx1^Cre^*^/+^*;Wt1^loxp^*^/+^ mice were crossed with female *Wt1^loxp/loxp^* mice to inactivate *Wt1* conditionally using *Prx1-Cre*. No live mutants (*Prx1^Cre^*^/+^*;Wt1^loxp/loxp^*) were present when the litters were genotyped at ∼2-3 weeks of age (*n*>50). Given the important role of *Wt1* in regulating key developmental processes (Chau and Hastie, 2012), we suspected that the phenotypes of the mutants probably resulted in embryonic lethality. However, mutant embryos appeared to be grossly normal (externally) at all stages analysed (E11.5, E12.5, E14.5, E16.5, E18.5 and E19.5). The number of mutant embryos obtained at each stage is summarised in Table S1. When the pregnant dams were left to give birth, it was apparent that mutant pups were born alive but died within a few hours. Obtaining mutant mice that survived until birth led us to hypothesise that their death might have been caused by an inability to breathe. Diaphragmatic defects typically result in disrupted breathing (Greer, 2013). As mentioned previously, *Wt1* null mouse embryos also develop diaphragmatic hernias (Kreidberg et al., 1993). Therefore, we hypothesised that the *Prx1^Cre^*^/+^*;Wt1^loxp/loxp^* embryos might have diaphragmatic hernias.

**Fig. 1.**
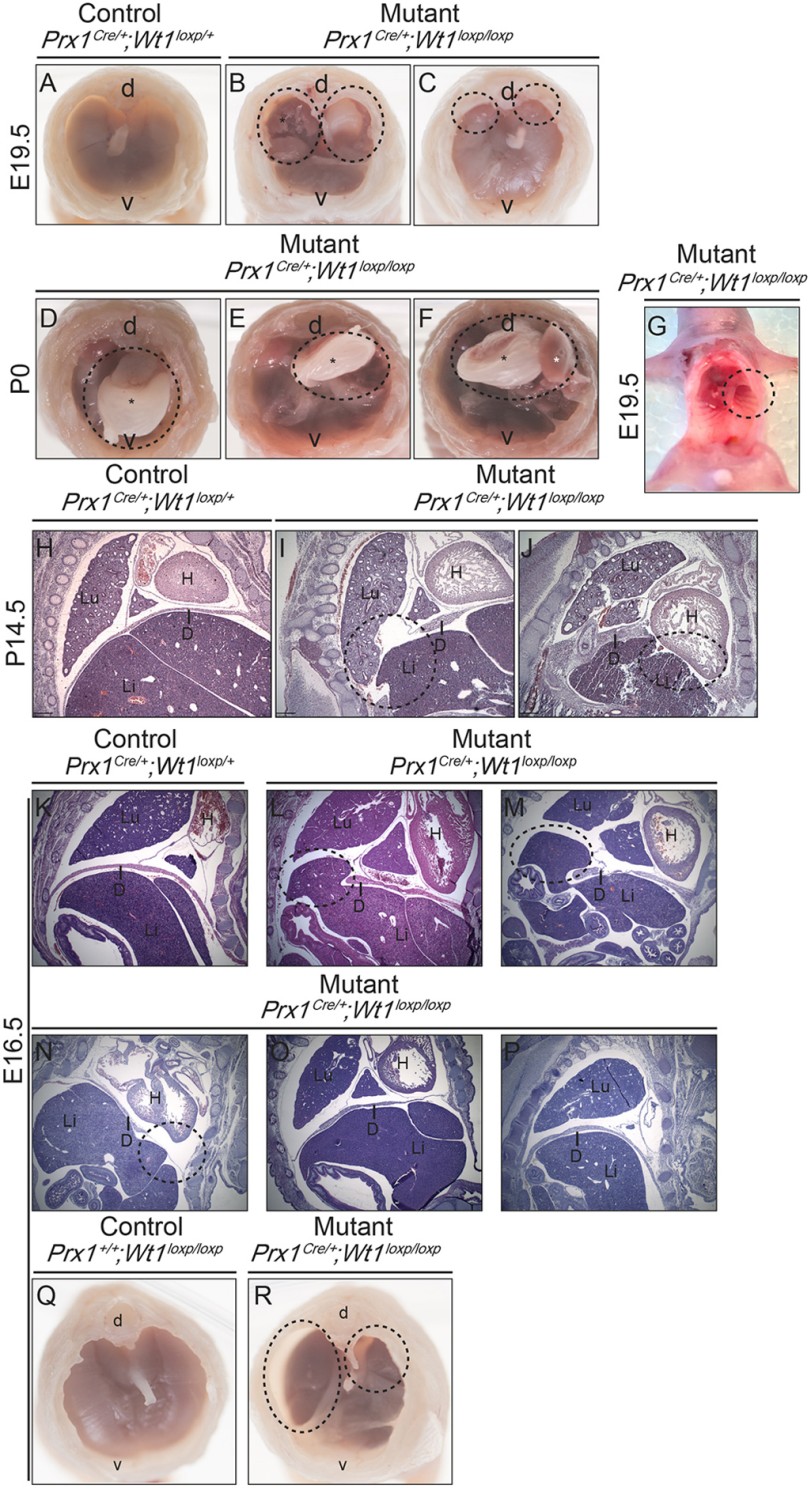
**Deletion of *Wt1* using *Prx1-Cre* leads to mutant embryos and pups with diaphragmatic hernia (*Prx1^Cre^*^/+^*;Wt1^loxp/loxp^*).** Male *Prx1^Cre^*^/+^*;Wt1^loxp^*^/+^ mice were crossed with female *Wt1^loxp/loxp^* mice. Embryos from various stages or pups (which died shortly after being born) were taken for analysis. Mutants (*Prx1^Cre^*^/+^*;Wt1l^oxp/loxp^*) exhibited diaphragmatic hernia. (A-G) Pups were dissected, heads and thoracic content removed, and the diaphragm was imaged from the top. (B,C) Images of hernias in the E19.5 mutant embryos. (B) Bilateral and dorsally located holes were found in the diaphragm (circled), with liver herniation into the thoracic cavity (asterisk). (C) Smaller bilateral and dorsally located holes were observed (circled), and a littermate control is shown in A. (D-F) Representative images of hernias in the P0 mutant pups. Large and left-sided dorsally located holes (circled), with herniation of the stomach (black asterisks) and liver herniation (white asterisk). (G) An E19.5 mutant embryo imaged from below with abdominal content removed, revealing a left-sided dorsally located hole in the diaphragm (circled). (H-P) E14.5 (H-J) and E16.5 (K-P) embryos were sectioned and stained with H&E. Holes in the diaphragm are circled. Representative immunohistochemistry images from a minimum of three control and three mutant samples are shown. (Q,R) Freshly isolated E16.5 embryos were dissected, contents of the thoracic cavity removed, and they were imaged from the anterior of the embryo. (R) Bilateral dorsal holes were present in the mutant embryo (circled). D, diaphragm; d, dorsal; H, heart; Li, liver; Lu, lung; v, ventral.

We analysed deceased [postnatal day (P) 0] and E19.5 mutant embryos and found large holes in their diaphragms (Fig. 1A-G). Younger mutant embryos (E14.5 and E16.5) were also found to have diaphragmatic holes (Fig. 1H-J and K-R, respectively), often accompanied by liver herniation into the thoracic cavity (Fig. 1I,J,L,M). Between 80% and 90% of CDH is Bochdalek type, characterised by hernias in the posterolateral region of the diaphragm. In more than 85% of cases, Bochdalek hernias are left sided (Longoni et al., 2006). A description of the phenotypes of the *Prx1^Cre^*^/+^*;Wt1^loxp/loxp^* embryos at different stages is summarised in Table S2, with the left-sided hernia being the most common defect (45%). Twenty percent of the mutants had bilateral hernias, and 5% had holes on the right side only. Finally, 30% of the mutants either did not exhibit any obvious diaphragmatic phenotype or did not have fully formed holes, but a thinning of the diaphragm was observed.

**Fig. 2.**
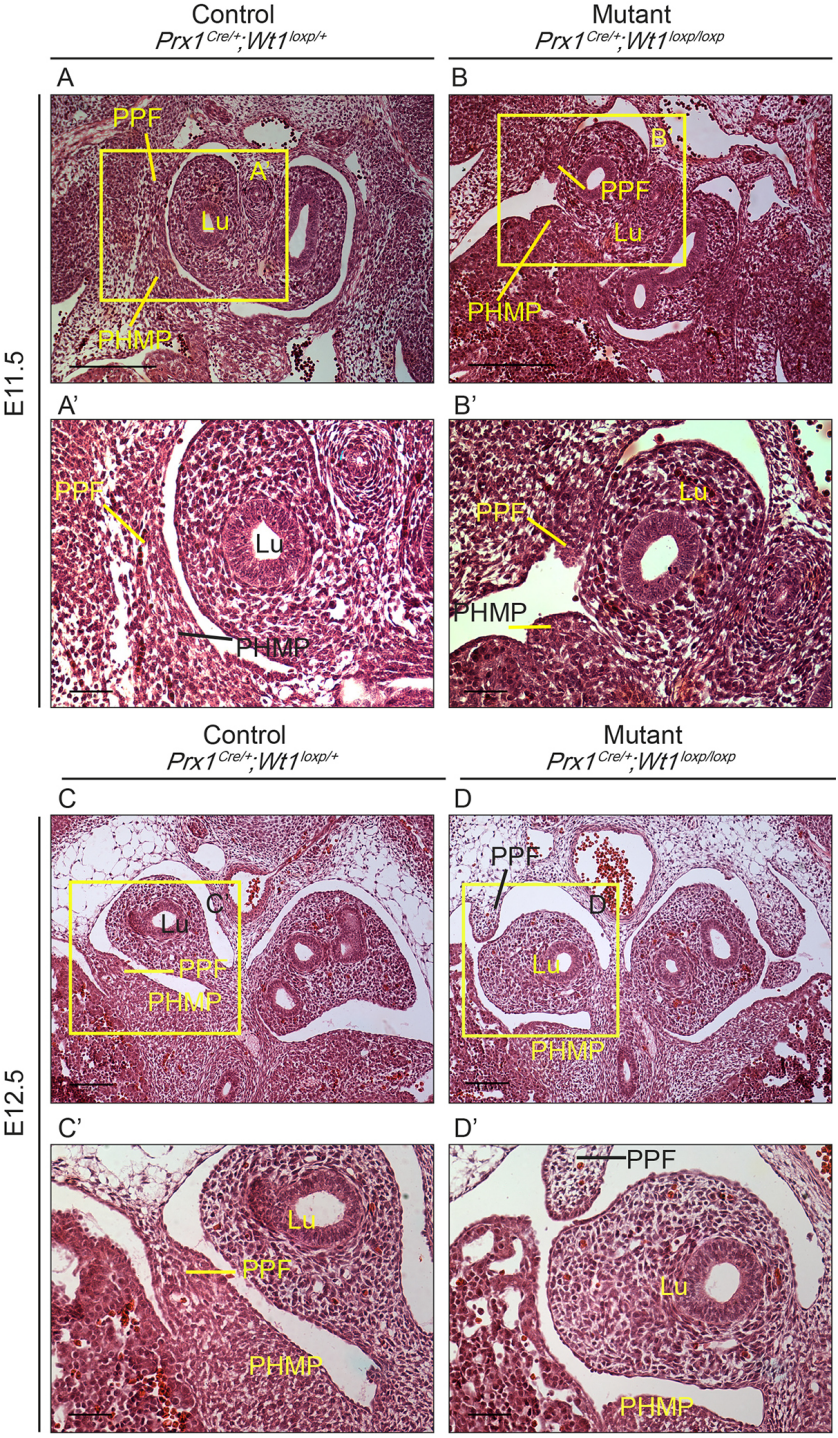
***Prx1^Cre^*^/+^*;Wt1^GFP/loxP^* embryos have disrupted PPF formation.** (A,B) Representative images of E11.5 H&E-stained sections are shown, illustrating disrupted PPF development in the mutants (B). A gap is visible between PPF and PHMP. A littermate control is shown in A. (A′,B′) Magnified images of the areas boxed in A,B. (C,D) Representative images of E12.5 H&E-stained sections are shown. (D) Images of disrupted PPF development. (C) Images of a littermate control, showing a continuous band of PPF/PHMP. (C′,D′) Magnified images of the areas boxed in C,D. Representative immunohistochemistry images from a minimum of three control and three mutant samples are shown. Lu, lungs; PHMP, posthepatic mesenchymal plate; PPF, pleuroperitoneal fold. Scale bars: 100 µm in A-D; 50 µm in A′-D′.

The initial separation of the thoracic and peritoneal cavities is established by the PPFs, which are typically formed between E10.5 and E12.5 (Carmona et al., 2016; Merrell et al., 2015). By E12.5, a continuous band of cells has developed, completely separating the two cavities. Haematoxylin and Eosin (H&E)-stained sections of E11.5 and E12.5 mutant embryos (Fig. 2A-D′, sectioned transversely) showed that this continuous band of cells failed to form correctly (Fig. 2B,B′,D,D′). Large gaps between the PPFs and the PMPH were observed in the mutants (Fig. 2B,B′,D,D′), leading to the failure of separation of the two cavities.

### *Prx1-Cre* labelling of the WT1^+^ mesenchymal cells in the PPFs

**Fig. 3.**
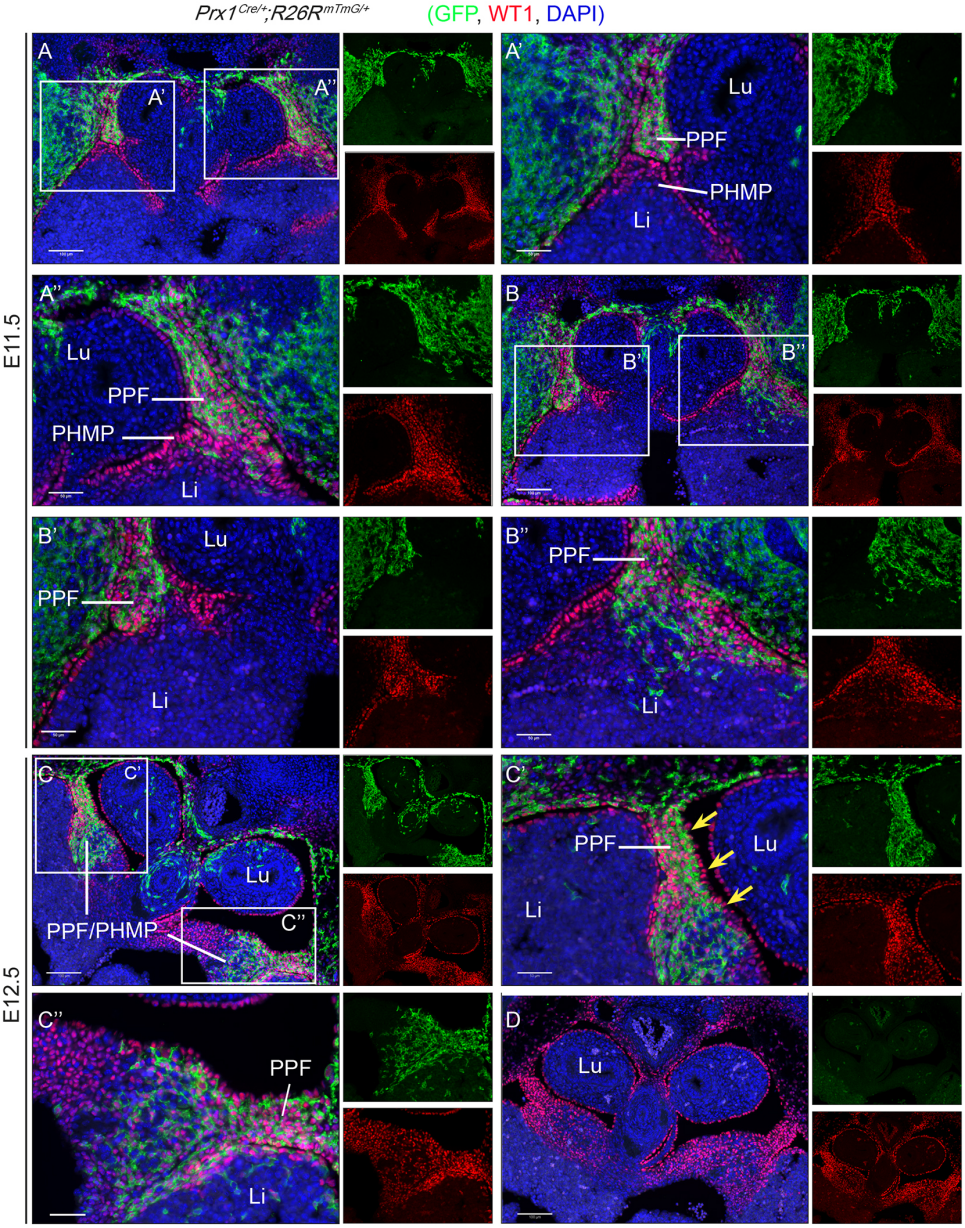
**The PPFs, but not the PHMPs, are *Prx1-Cre* lineage positive.** Transversely sectioned E11.5 or E12.5 *Prx1-Cre* lineage-tracing embryos (*Prx1^Cre^*^/+^*;R26R^mTmG^*^/+^) are stained with an anti-GFP antibody (indicated in green) and an anti-WT1 antibody (red). Cell nuclei are stained with DAPI (blue). (A,B) Consecutive sections, where B is anterior to A. WT1 expression (red) is detected in both PPFs and PHMPs, whereas GFP signal is detected only in PPFs. (B″) The expansion of PPFs into the PHMPs, starting to form a continuous band. (C) This PPF/PHMP continuum is fully formed at E12.5. GFP-expressing cells are located at the edge where the PPF and PHMP are merged/fused. The mesothelial cells of PPFs do not express GFP (yellow arrows in C′). (A′-C″) Magnified images of the areas in A-C. (D) A *Prx1-Cre*-negative littermate control. Li, liver; Lu, lungs; PHMP, posthepatic mesenchymal plate; PPF, pleuroperitoneal fold. Scale bars: 100 µm in A-D; 50 µm in A′-C″. *n*=4 animals.

To understand the cause of this defect, we first needed to know the location of *Prx1-Cre* expression (i.e. in which cells *Wt1* was being deleted). Immunofluorescence was performed on sections from *Prx1^Cre^*^/+^*;R26R^mTmG/+^* lineage-tracing embryos using anti-green fluorescent protein (anti-GFP) and anti-WT1 antibodies. In this model, cells in which *Prx1-Cre* is or has been expressed are GFP^+^. As shown in Fig. 3, WT1 expression was detected in the PHMP and PPFs (indicated in red). Although the cells of the PPFs expressed GFP and were thus *Prx1-Cre* lineage positive (indicated in green), no expression of GFP was detected in the cells of the PHMP (Fig. 3A,A′). This was particularly obvious in the E11.5 embryos. At E11.5, the PPFs and PMHPs have begun to fuse, with boundaries still clearly visible between the two structures. This boundary is distinctly marked by the clear domains of GFP-expressing cells. To demonstrate this better, consecutive sections in this region were obtained and stained (Fig. 3A,B, where B is anterior to A). In Fig. 3A′,A″, the PPFs (GFP^+^) and PHMPs (GFP^−^) can be seen abutting one another, whereas in Fig. 3B′,B″, it is clear that the PPFs (GFP^+^) are merging with the PHMPs (GFP^−^). At E12.5, once the continuous band has been formed, the population of GFP^+^ cells (PPF derived) infiltrating the PHMP is still distinctive (Fig. 3C).

Like other visceral organs, the diaphragm is lined by a mesothelial layer. The mesothelial cells of the diaphragm express endogenous WT1 (as indicated by the yellow arrows in Fig. 3C′); however, this mesothelial layer does not express GFP and therefore is not derived from the *Prx1-Cre* lineage and thus not from the PPFs. Using this model, we have demonstrated that only the PPFs arise from *Prx1-Cre*-expressing cells and not the PHMP. Therefore, despite WT1 being expressed in both the PPFs and PHMP, it will be deleted only in the cells of the PPFs in our model.

**Fig. 4.**
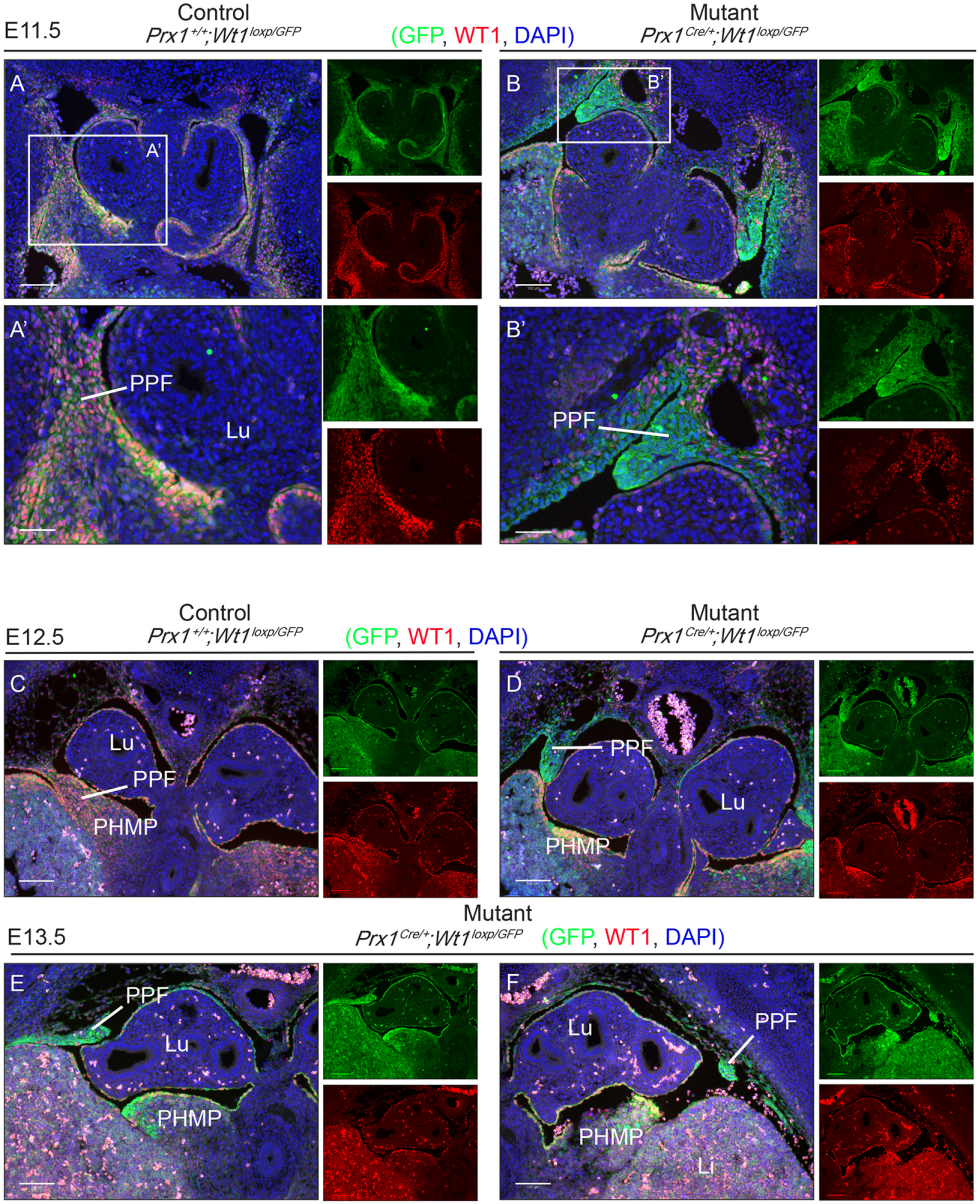
***Wt1* deletion in the PPFs leads to disrupted PPF development in the *Prx1^Cre^*^/+^*;Wt1^GFP/loxP^* embryos, where presence of GFP expression and absence of WT1 expression indicate cells with *Wt1* deletion.** (A-F) Representative images of E11.5 (A-B′), E12.5 (C,D) and E13.5 (E,F) sectioned embryos. Slides were stained with an anti-GFP antibody (green; upper inset), an anti-WT1 antibody (red; lower inset) and DAPI (blue). (B,D-F) Images from mutant embryos, where disrupted PPF expressed GFP but not WT1, indicating deletion in this structure. (A,C) A *Prx1-Cre*-negative littermate control from each stage is shown. (A′,B′) Magnified images of areas indicated in A,B. Lu, lungs; Li, liver; PHMP, posthepatic mesenchymal plate; PPF, pleuroperitoneal fold. Scale bars: 100 µm in A-F; 50 µm in A′,B′. *n*=3 animals for each genotype.

To illustrate this better, a second mouse model was generated. *Prx1^Cre^*^/+^*;Wt1^GFP^*^/+^ males were crossed with *Wt1^loxP/loxP^* females. In *Prx1^Cre^*^/+^*;Wt1^loxP/GFP^* offspring, one copy of the *Wt1* allele is flanked by loxP sites, whereas the other copy has a GFP knock-in at exon 1, which disrupts function. Therefore, WT1-expressing cells will be GFP^+^. Moreover, upon Cre-mediated loxP recombination (driven by *Prx1-Cre*), the second copy of *Wt1* is conditionally deleted. The mutant cells remain GFP^+^ despite no functional WT1 being present. Staining with anti-GFP and anti-WT1 antibodies reveals cells that have once expressed WT1 but no longer do so. Such cells will be positive for GFP but negative for endogenous WT1. This is illustrated in Fig. 4 (E11.5, E12.5 and E13.5), where endogenous WT1 expression (red) was detected in PPFs and PHMP in the control embryos (Fig. 4A,A′,C), but its expression was completely lacking in the PPFs and unaffected in the PHMP of the mutant embryos (Fig. 4B,B′,D,F). GFP expression was observed in the disrupted PPFs, thus confirming that WT1 would normally be expressed in these cells (Fig. 4B,B′,D-F). The presence of these GFP^+^ cells also suggests that *Wt1* deletion in these cells did not lead to cell loss. Intriguingly, the GFP signal in the PPFs of the mutant embryos was much stronger than that in the control littermates (Fig. 4). It is plausible that this was a consequence of an accumulation of cells in the PPFs owing to defective cell movement. Identifying PPF and PHMP structures is not trivial, because sections have to be obtained at precisely the same level from control and mutant embryos. This model provides a reassuring way of identifying the regions in which *Wt1* has been deleted, which is convenient for subsequent studies of pathways that are potentially disrupted.

### The epithelial status of PPFs with *Wt1* deleted is unchanged

Previously, we have shown that *Wt1* plays a major role in regulating the epithelial and mesenchymal cell states in several mesodermal tissues (Martínez-Estrada et al., 2010). To test the hypothesis that the formation of the continuous band of cells is disrupted owing to defects in PPF cell migration, we analysed the expression levels of markers for epithelial-to-mesenchymal transition (EMT). We stained sections from control (*Prx1^+^*^/+^*;Wt1^GFP/loxP^*) and mutant (*Prx1^Cre^*^/+^*;Wt1^GFP/loxP^*) embryos with an anti-E-cadherin (CDH1; an epithelial marker) antibody and an anti-vimentin (VIM; a mesenchymal marker) antibody. Sections were co-stained with an anti-WT1 antibody or an anti-GFP antibody to demonstrate deletion of *Wt1* and regions of PPFs and PHMPs. Despite strong expression of CDH1 in the lung bud (a positive control), no expression of CDH1 was detected in the PPFs of the mutant or control embryos (Fig. S1C-D′; CDH1 is indicated in green and GFP in red). Therefore, we did not observe any change in CDH1 expression in the PPFs between the control and mutant embryos. We also compared expression of VIM between control and mutant embryos (VIM is indicated in green and WT1 in red), but no changes were observed (Fig. S1A-B′). Next, we investigated whether cell proliferation within the mutant PPFs was reduced. We co-stained sections with anti-Ki67 (also known as Mki67) and anti-GFP antibodies (Fig. S2; Ki67 is indicated in red and GFP in green). Quantification of the percentage of cells that are positive for Ki67 expression suggests no significant differences between control and mutant embryos (*n*=3 animals for each genotype, Mann–Whitney *U*-test, two tailed, *P*-value>0.5). Ki67 marks nuclei in all active phases of the cell cycle. Inclusion of a more specific mitotic marker, such as phosphohistone H3, can provide a more reliable indication about whether the lack of a continuous band of cells is caused by a disruption in PPF cell proliferation.

The retinoic acid (RA) signalling pathway plays a key role in development of the diaphragm. A common mouse model of CDH uses nitrofen to inhibit RA synthesis (Greer et al., 2003; Mey et al., 2003). We analysed the expression of RALDH2 (also known as ALDH1A2), which catalyses the formation of RA from retinaldehyde. Importantly, WT1 has been shown to activate *Raldh2* transcriptionally in epicardial cells (Guadix et al., 2011). Surprisingly, we observed an increase in RALDH2 expression in the PPFs of the mutant embryos (Fig. S3B,B′). The increase in RALDH2 expression in the mutant rudiments is likely to be attributable to individual cells expressing higher levels of RALDH2. Mutant cells in the affected area expressed higher levels of RALDH2 per cell (*n*=4 animals) compared with the control cells (*n*=3 animals, Mann–Whitney *U*-test, two tailed *P*-value=0.05; Fig. S3E), quantified by measurement of the RALDH2 immunofluorescence intensity.

To understand plausible molecular pathways that might be underlying the defects in our model, we generated a mouse model to allow the precise isolation of cells in the diaphragmatic region in which *Wt1* has been deleted (*Prx1^Cre^*^/+^*;R26R^tdRFP^*^/+^*;Wt1^loxP/GFP^*). In this model, cells that are derived from the *Prx1-Cre* lineage and have had *Wt1* deleted will express both GFP and red fluorescent protein (RFP). We used fluorescence-activated cell sorting (FACS) to sort these cells from the diaphragmatic region of E11.5 embryos. Control cells were sorted from the same region isolated from a comparable model, differing only by the absence of loxP sites flanking one of the *Wt1* alleles (*Prx1^Cre^*^/+^*;R26R^tdRFP^*^/+^*;Wt1*^+/*GFP*^), therefore avoiding the deletion of *Wt1* in the *Prx1-Cre* lineage. We measured expression levels of known EMT markers in these cells, including snail family zinc finger 1 (*Snai1*) and 2 (*Snai2*). Fig. S4 shows that the levels of these EMT markers are not altered, which is consistent with the CDH1 and VIM results shown in Fig. S1.

### PPFs give rise to non-muscle mesenchyme, and muscle connective tissue fibroblasts and myoblasts are absent in mutant PPFs

**Fig. 5.**
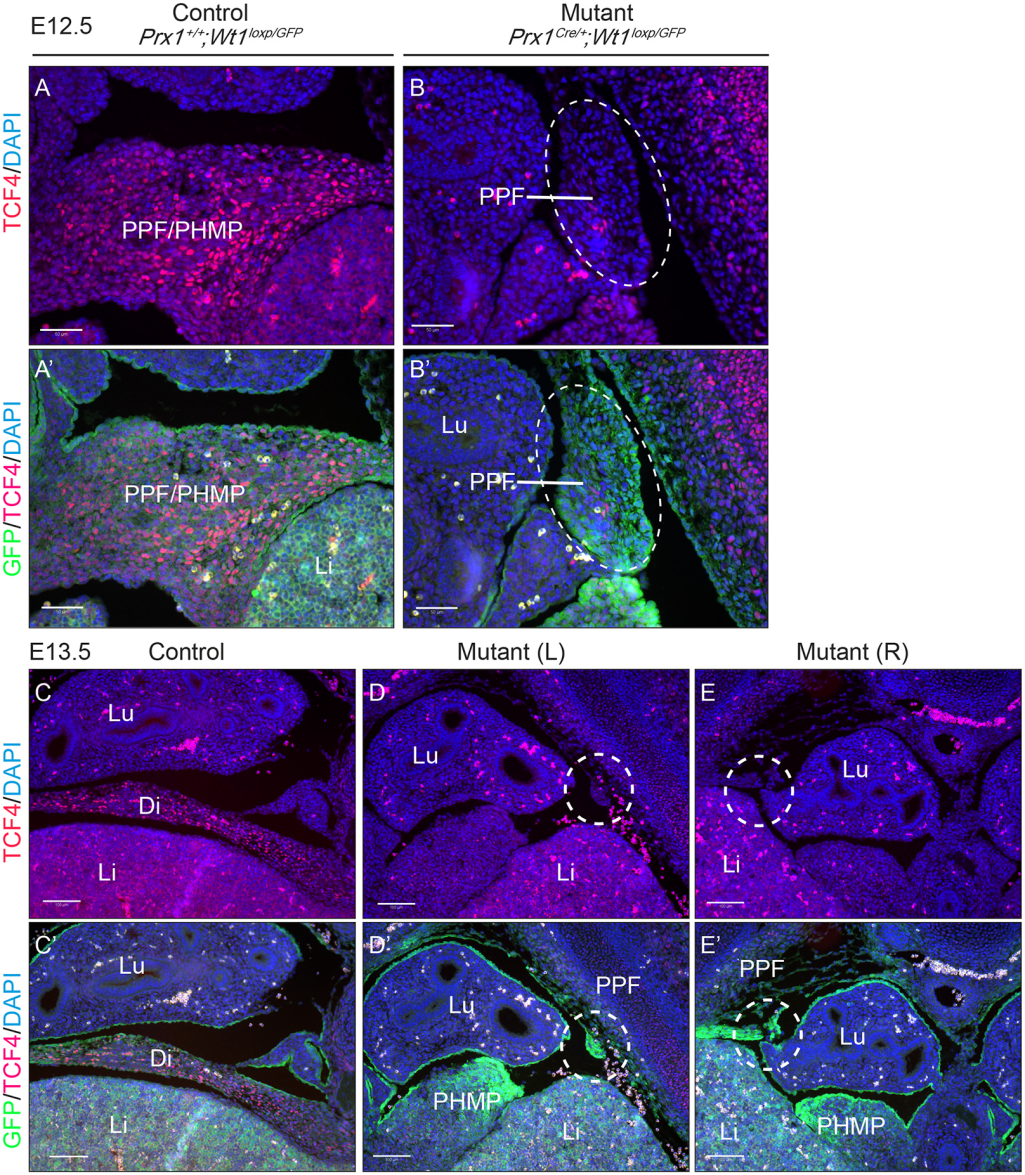
***Wt1* deletion in the PPFs leads to absence of TCF4-expressing cells in the PPFs of *Prx1^Cre^*^/+^*;Wt1^GFP/loxP^* embryos.** Representative images of E12.5 and E13.5 embryos were sectioned and stained with an anti-TCF4 antibody (red), anti-GFP antibody (green) and DAPI (blue). (A-B′) At E12.5, TCF4-expressing cells are located in the PPF/PHMP continuum in the control embryo (A,A′), whereas these cells are absent in the mutant embryos (B,B′). (C-E) At E13.5, TCF4-expressing cells (red) are located in the diaphragm of the control embryos (C,C′) and absent in the disrupted PPFs/PHMPs in the mutant embryos (D-E′). The left (L) side of disrupted PPF/PHMP is shown in D,D′ and the right (R) side in E,E′. (A′-E′) Merged images of areas indicated in A-E. Di, diaphragm; Li, liver; Lu, lungs; PHMP, posthepatic mesenchymal plate; PPF, pleuroperitoneal fold. Scale bars: 50 µm in A-B′; 100 µm in C-E′. *n*=3 animals for each genotype.

Defects in the PPFs have been suggested to be the cause of CDH in several mouse models (Merrell et al., 2015). The PPFs have been shown to give rise to the central tendon and the muscle connective tissue fibroblasts, characterised by TCF4 and GATA4 expression (Merrell et al., 2015). In addition, work performed by Paris et al. (2016) shows that WT1-expressing cells in the PPFs are non-muscle mesenchymal cells, and the majority of them do not express TCF4. This is supported by our data showing that the majority of WT1^+^ cells (indicated by GFP staining, green) do not express TCF4 (indicated in red; Fig. 5A′). Quantification shows that 6.7% of WT1^+^ cells express TCF4 (s.d.=0.9%, *n*=3 animals). Together, these results suggest that the TCF4-expressing cells (muscle connective tissue fibroblasts) and the WT1-expressing cells in the PPFs are likely to be two distinct cell types. In addition to the central tendon and the muscle connective tissue fibroblasts, our data suggest that the PPFs also give rise to the non-muscle mesenchyme (marked by WT1).

As PPFs expand, connective tissue fibroblasts (TCF4^+^ and GATA4^+^) guide migration of myoblasts. We examined the expression of TCF4 in our model, in which *Wt1* is deleted in the non-muscle mesenchymal cells by *Prx1-Cre*. Unexpectedly, TCF4 expression (indicated in red) was almost completely absent in the mutant PPFs at both E12.5 and E13.5 (Fig. 5A-E). Single-channel images representing GFP expression are included in Fig. S5. Despite dissimilar PPF/PHMP appearance, the sections were taken at comparable levels between control and mutant embryos using their positions relative to the lung, liver and oesophagus for guidance. The positioning of PPF/PHMP relative to other organs is often used where 3D imaging of embryos is not readily accessible (Carmona et al., 2016; Coles and Ackerman, 2013; Domyan et al., 2013; Paris et al., 2016; Wat et al., 2012). In mutant embryos, defective PPFs appear as residual rudiments, whereas PPF and PHMP have already formed a continual band in control embryos (E12.5 and E13.5). This absence of TCF4^+^ cells in mutant PPF rudiments was not expected, because WT1 is not expressed in the muscle connective tissue fibroblasts in PPFs (Paris et al., 2016), suggesting plausible interactions between these two populations of cells.

**Fig. 6.**
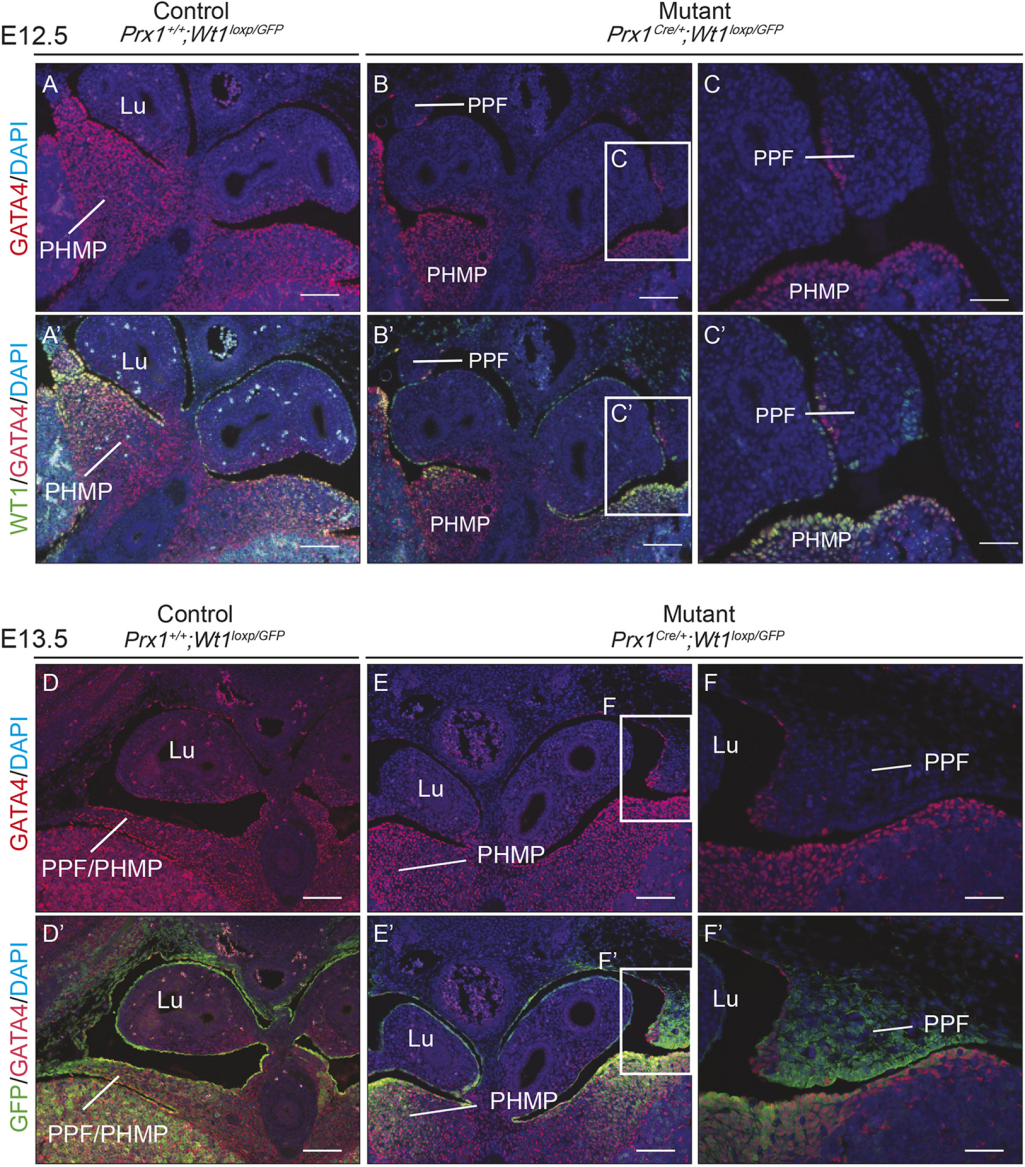
***Wt1* deletion in the PPFs leads to absence of GATA4-expressing cells in the PPFs of *Prx1^Cre^*^/+^*;Wt1^GFP/loxP^* embryos.** (A-C′) Representative images of E12.5 embryos sectioned and stained with an anti-GATA4 antibody (red), an anti-WT1 antibody (green) and DAPI (blue). Images from mutant embryos are shown in B-C′, where WT1 expression in the PPF is deleted and the GATA4-expressing cells are absent in the PPFs. Images from a *Prx1-Cre*-negative littermate control are shown in A,A′. Boxed areas in B,B′ are magnified in C,C′. *n*=3 animals for each genotype. (D-F′) Representative images of E13.5 embryos sectioned and stained with an anti-GATA4 antibody (red), an anti-GFP antibody (green) and DAPI (blue). Representative images from *Prx1-Cre*-negative littermate controls are shown in D,D′. Images of mutant embryos showing disrupted PPF development are shown in E,E′. (F,F′) Magnified images of boxed areas in E,E′. *n*=2 animals for controls and *n*=3 animals for mutants. Lu, lungs; PHMP, posthepatic mesenchymal plate; PPF, pleuroperitoneal fold. Scale bars: 50 µm in A-B′,D-E′ 100 µm in C,C′,F,F′.

In addition to labelling muscle connective tissue fibroblasts, TCF4 is a transcription factor that binds to B-catenin (CTNNB1) (Korinek et al., 1997) in developing diaphragm (Paris et al., 2016). The Wnt/CTNNB1 signalling pathway is also known to act downstream of WT1 (Kim et al., 2009). The reduction in TCF4 expression in the mutant PPFs in our model could therefore be attributable to the disruption of the Wnt/CTNNB1 pathway as a result of reduced *Wt1* expression. Alternatively, the disappearance/absence of muscle connective tissue fibroblasts, which are marked by TCF4, could also explain the reduction. To test the second possibility, sections were stained with an anti-GATA4 antibody, which also marks muscle connective tissue fibroblasts (Merrell et al., 2015). At E12.5, GATA4 expression was found at high levels in cells of the PHMP and in the continuous band of cells separating the cavities (Fig. 6A,A′). The expression was particularly strong in the mesothelium of the PPFs but much weaker (if not absent) in the mesenchymal/fibroblast cells within the PPFs (Fig. 6A,A′). As with the TCF4 staining, no GATA4-expressing cells were found in the PPFs in which *Wt1* was deleted (Fig. 6B-C′; E12.5 embryos from the *Prx1^Cre^*^/+^*;Wt1^loxP/GFP^* model, where GATA4 is indicated in red and WT1 and/or GFP in green). We also checked GATA4 expression at E13.5. Likewise, no GATA4 expression was detected in the rudiment of the PPFs in the mutant embryos (Fig. 6E-F′) compared with clear expression in the controls (Fig. 6D,D′), suggesting an absence of muscle connective tissue fibroblasts in the mutant embryos. Single-channel images representing WT1 and GFP expression are shown in Fig. S6.

**Fig. 7.**
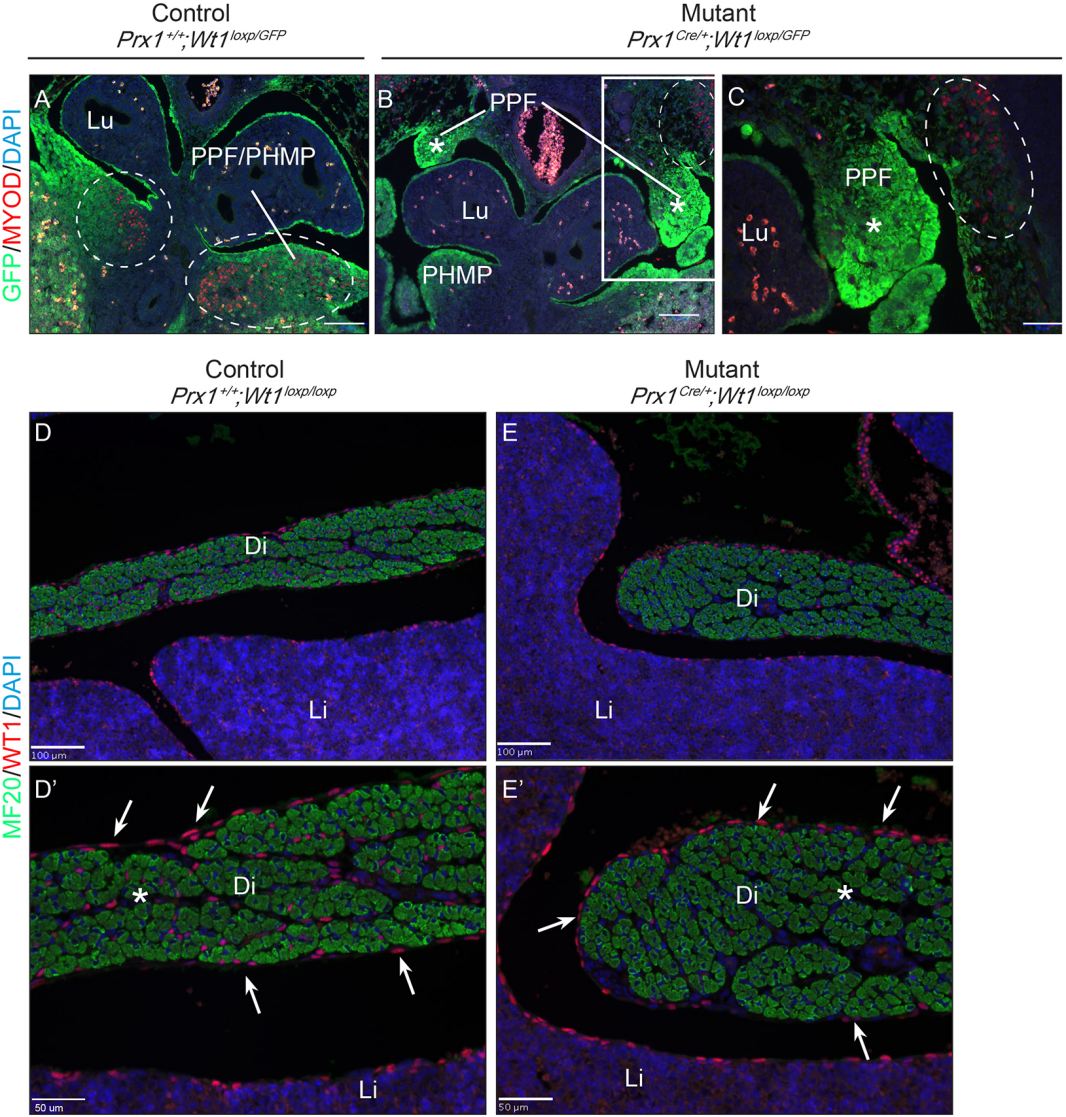
***Wt1* deletion in the PPFs leads to absence of MYOD-expressing myoblasts in the PPFs of *Prx1^Cre^*^/+^*;Wt1^GFP/loxP^* embryos.** Representative images of E12.5 embryos were sectioned and stained with an anti-MYOD antibody (red), anti-GFP antibody (green) and DAPI (blue). (A) Images from control embryos, showing myoblasts (red, circled) located in the PPF/PHMP continuum. (B,C) In the mutant embryos, myoblasts are absent in the disrupted PPF/PHMP (white asterisks in B,C). Boxed area in B is magnified in C. Circled area indicates MYOD^+^ cells located in the lateral body wall. (D-E′) E16.5 embryos sectioned in the sagittal plane were stained with an anti-MF20 (green) and an anti-WT1 antibody (red). WT1 expression is lost in the mesenchymal cells of the diaphragm (white asterisks) but not in the mesothelial cells (white arrows). Parts of the areas in D and E are magnified in D' and E', respectively. Di, diaphragm; Li, liver; Lu, lungs; PHMP, posthepatic mesenchymal plate; PPF, pleuroperitoneal fold. Scale bars: 100 µm in A,B,D,E; 50 µm in C,D′,E′. Together, *n*=3 animals for controls and *n*= 6 animals for mutants were analysed by MF20 staining.

The GATA4- and TCF4-expressing muscle connective tissue fibroblasts have been shown to play a crucial role in guiding the migration of myoblasts (Merrell et al., 2015). Myogenic progenitors begin to migrate from the somites to the diaphragm at around E12.5 (Merrell and Kardon, 2013). To test whether the myoblasts were affected in our model, we stained sections with an anti-MYOD (also known as MYOD1) antibody. In the control E12.5 diaphragm, MYOD^+^ cells were clearly visible in the PPF/PHMP continuum (indicated in red; Fig. 7A), whereas there was a complete absence of MYOD^+^ cells in the mutant PPF rudiments (Fig. 7B-C′). MYOD^+^ cells were also absent in the mutant PHMPs, because PPFs failed to reach PHMPs to form the PPF/PHMP continuum. The myoblasts were present in the lateral body wall, suggesting that their formation was not affected (Fig. 7B,C; myoblasts in lateral body wall are circled). In the mutant diaphragm at E16.5, there was muscle formation. Additionally, a thickening of the diaphragm was observed at the edges of the hernia, consistent with previous reports of disrupted migration in other models of CDH (Clugston et al., 2006). Sections were stained with an anti-MF20 antibody, which detects the heavy chain of myosin II, indicated in green, and WT1 is indicated in red (Fig. 7D-E′). Single-channel images are included in Fig. S7. WT1 expression was lost in the mesenchymal cells of the diaphragm (white asterisk) but not in the mesothelial cells (white arrows). These data suggest that deletion of *Wt1* in the non-muscle mesenchyme results in an absence of muscle connective tissue fibroblasts owing to unknown mechanisms and a failure of myoblast migration to the PPFs, ultimately leading to disrupted diaphragm formation.

## DISCUSSION

CDH is a complex condition, and many aspects of this disease are poorly defined, particularly the underlying causal mechanisms. The formation of diaphragm is a delicate process that involves multiple cell types arising from several regions (somites, lateral plate mesoderm, neurons and ST). Different cell types interact with one another (for example, muscle connective tissue fibroblasts guide the migration of the myogenic progenitors; Merrell et al., 2015), and crosstalk between signalling pathways is common. The complexity of diaphragm development no doubt contributes to the complex aetiology of CDH. Here, we use transgenic mouse models to dissect the role of one crucial gene, *Wt1*, in the mesenchymal cells of the PPFs.

Initially, we demonstrate the heterogeneous nature of the mesenchymal cell populations within the PPFs. As described previously, *Prx1-Cre* (expressed in the lateral plate mesoderm during development) gives rise to muscle connective tissue fibroblasts, marked by TCF4 and GATA4 (Merrell et al., 2015). In agreement with a previous study (Paris et al., 2016), we show that most of the TCF4^+^ (and GATA4^+^) cells in the PPFs do not express WT1. This suggests that in addition to the muscle connective tissue fibroblasts (Merrell et al., 2015), *Prx1-Cre*-expressing cells give rise to another cell type in the developing diaphragm, with mesenchymal properties (marked by WT1). We next show that these WT1-expressing PPF cells (from the *Prx1-Cre* lineage) have the ability to expand and form a continuous band of cells with PHMP, ultimately sealing off the thoracic and peritoneal cavities. It is interesting to note that in the CDH model in which *Gata4* is deleted in the muscle connective tissue fibroblasts using *Prx1-Cre* (*Prx1Cre^Tg^*^/+^;*Gata4*^∇*/flx*^*;Rosa26^LacZ^*^/+^), B-gal^+^ sacs are present covering herniated regions, and not as holes in this tissue (Merrell et al., 2015). Given that our data demonstrate that cells in the *Prx1-Cre*-expressing lineage can give rise to mesenchymal cells that are able to expand, it is likely that these mesenchymal cells might contribute to the thin membranous sac formed in the model described by Merrell et al. (2015). However, in our model, in which *Wt1* is deleted in mesenchymal cells of the PPFs using *Prx1-Cre*, we observe a failure of closure of the body cavities at early stages and holes/hernia at later stages. Together, these results suggest that the WT1-expressing mesenchymal cells of the PPFs might be one driving force behind expansion of PPFs towards the PHMP.

Moreover, our data suggest that cells of the PHMP might also move towards the PPFs. In the diaphragms of E12.5 embryos (at which point the continuous PPF/PHMP membrane is almost complete), GATA4 expression is high in the cells of the PHMP, and most of these GATA4^+^ cells co-express WT1. Our staining suggests that the GATA4^+^ cells in the PHMP migrate towards the PPFs, because most of the cells in the PPFs do not express GATA4. This forms a distinct boundary at the point at which the PPFs (GATA4^−^) and PHMP (GATA4^+^) meet. A similar staining pattern has been described in a recent study in which G2-*Gata4-Cre* was used to delete *Wt1* (Carmona et al., 2016).

**Fig. 8.**
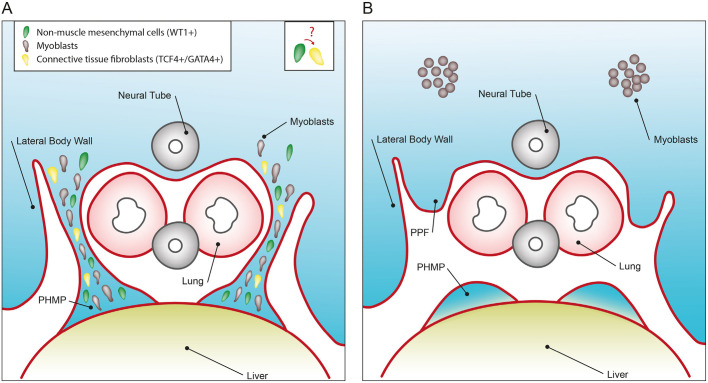
**A cartoon illustration of the formation of the diaphragm.** Data from our model suggest the heterogeneity of mesenchymal cells in the PPFs and the importance of the WT1-expressing non-muscle mesenchyme for development of the diaphragm. (A,B) Normal (A) and mutant condition (B), in which *Wt1* is conditionally deleted by *Prx1-Cre*. We demonstrated that the non-muscle mesenchymal cells (indicated in green) in the PPFs expand and merge with PHMPs to form a continuous band. Deletion of *Wt1* using *Prx1-Cre* leads to a pause of these mesenchymal cells and formation of a gap. In the PPFs with deletion of *Wt1*, connective tissue fibroblasts (indicated in yellow) are absent for unknown reasons. We speculate a possible crosstalk between non-muscle mesenchymal cells and the connective tissue fibroblasts. Absence of connective tissue fibroblasts might lead to the disruption of myoblast (indicated in brown) migration to the PPFs.

Connective tissue fibroblasts (indicated in yellow in the cartoon illustration; Fig. 8) are absent from the PPFs in our model, in which *Wt1* is deleted in the non-muscle mesenchymal cells (indicated in green in the cartoon illustration; Fig. 8) using *Prx1-Cre*. Interactions between two cell types are common. For example, in skeletal muscle, ablation of muscle satellite cells (PAX7^+^) severely reduces the expansion of muscle connective tissue fibroblasts (TCF4^+^) during regeneration (Mathew et al., 2011). The molecular mechanisms that might govern the interactions between these cell types during development of the diaphragm remain incompletely understood (cartoon illustration; Fig. 8).

In addition to connective tissue fibroblasts (yellow; Fig. 8) and non-muscle mesenchymal cells (green; Fig. 8), there is an additional player in diaphragm development, the myoblasts (brown; Fig. 8). Absence of connective fibroblasts has previously been shown to cause defects in guiding the myogenic progenitors towards PPFs (Merrell et al., 2015). Staining with a muscle progenitor marker, MYOD, confirmed that this is also the case in our model. It should be noted that muscle fibres are present in the mutant diaphragm at later stages (e.g. E16.5), with possible disrupted orientation. In addition, shorter and thicker segments of diaphragm are present adjacent to the hernia in our mutant embryos at later stages. It is possible that muscle progenitors still retain some ability to move into the PPFs (or ‘ride on’ other moving cells as the embryo grows and expands in size), albeit less effectively, in the absence of the guiding connective tissue fibroblasts.

These data led us to investigate the underlying molecular mechanisms of the paused PPFs. Defects in EMT would be a plausible cause; however, we do not see changes in EMT markers at the protein (e.g. CDH1 and VIM by immunostaining) or mRNA level (e.g. *Snai1* and *Snai2* by quantitative PCR). An elegant study showed that conditional deletion of *Wt1* using a G2-*Gata4-Cre* results in the mice developing diaphragmatic hernias. Defects in EMT (as indicated in their study by an increase in CDH1, an epithelial cell marker) were suggested to be the underlying cause (Carmona et al., 2016). The Cre activity of the G2-*Gata4-Cre* is widely detected in lateral plate mesoderm at E7.75, but is then restricted in the ST and in visceral mesoderm at E9.5 (Rojas et al., 2005). At E10.5 and E11.5, the Cre activity of G2-*Gata4-Cre* is detected strongly in PHMP, with hardly any in the PPFs (Carmona et al., 2016; Rojas et al., 2005). In our model, we show that cells of the *Prx1-Cre* lineage are retained in PPFs and start to move from PPFs towards the PHMP. The difference between these results in EMT might be attributable to the different mouse models used; here, we delete *Wt1* in PPFs using *Prx1-Cre*, whereas the model described by Carmona et al. (2016) resulted in the deletion of *Wt1* predominantly in the PHMP. It is plausible that the cells that express *Wt1* in the PPFs differ from those that reside in the PHMP, because the two structures are derived from different regions during development.

Interestingly, we observed an increase in RALDH2 expression in the mutant PPFs deleted for *Wt1* in our model. We have shown previously that *Wt1* regulates the RA pathway by direct transcriptional activation of *Raldh2* in the epicardium (Guadix et al., 2011). This unexpected increase in RALDH2 in diaphragms with *Wt1* deleted has also been described in work performed by Carmona et al. (2016). In their model, the authors reasoned that the increase in RALDH2 was attributable to the intermediate mesoderm of the renal ridges persisting in the PPFs. *In situ* hybridization of *Pax2* (a marker of the intermediate mesoderm) provided evidence for their argument. However, no PAX2 expression was observed in the PPFs in our model (using an anti-PAX2 antibody; Fig. S3C). Clear PAX2 staining was observed in the neural tube, suggesting that the antibody worked well (Fig. S3D). Again, the difference between these results might be attributable to *Wt1* deletion using different Cre lines, hence affecting different regions or structures. Furthermore, RA is well known for its role in regulating the differentiation status of cells (Niederreither and Dollé, 2008), and RA levels have been shown to regulate the migration of cells during heart development (Wang et al., 2018). The elevated expression of RALDH2 seen in the mutant PPF rudiments where *Wt1* has been deleted could lead to changes in the differentiation status of cells in PPFs, and thereby affect their ability to expand.

*Wt1* plays crucial roles in diaphragm development, and its importance has been demonstrated in previous studies using mouse models. *Wt1* is expressed in several structures that are involved in diaphragm formation, including ST, PHMP, mesothelium, PPF and lateral body wall mesenchyme. Our model complements other studies by delineating the role of *Wt1* and the WT1^+^ mesenchymal cells by deleting *Wt1* in the PPFs and adjacent lateral body wall mesenchyme using *Prx1-Cre*. An early study, in 1993, in which *Wt1* was inactivated globally, resulted in embryonic lethality and multiple developmental defects, including defects in diaphragm formation (Kreidberg et al., 1993). This global *Wt1* knockout model was subsequently reanalysed and reported to have left-sided posterolateral defects (Clugston et al., 2006). A study performed by Paris et al. (2016) focused on the role of mesothelium in diaphragm development. Their *Wt1* loss-of-function experiments were carried out using homozygous *Wt1^GFPCre/GFPCre^* embryos (i.e. a global *Wt1* knockout). In our model, despite the strong expression of *Wt1* in mesothelium, we have shown clearly that there is no Cre activity detected in mesothelium using *Prx1-Cre*. In other words, mesothelium is not targeted in our model. As mentioned above, a conditional deletion of *Wt1* using G2-*Gata4-Cre* leads to *Wt1* deletion in ST and PHMP (Carmona et al., 2016; Rojas et al., 2005). *Wt1* deletion by G2-*Gata4-Cre* also leads to severe impairments of coronary vascular development and causes embryonic lethality around E15.5 (Cano et al., 2016). Interestingly, all the *Wt1* CDH models described above result in embryonic lethality, whereas our mice can survive until birth, and die shortly after owing to breathing difficulties. A left-sided dorsal defect is most frequent in our model (45%), followed by bilateral defects (20%) and thinning or no obvious phenotypes (15% each). The thinning of the diaphragm observed in some of our mutant embryos resembles diaphragmatic eventration, which is a rare condition that usually occurs as a result of defects/delays in the migration of myoblasts, leading to a structural deficiency of diaphragmatic muscle (Groth and Andrade, 2009).

About 25% of individuals with CDH also have abnormalities of one or more major body systems, and 50-60% of CDH cases are isolated, which means that affected individuals have no other major malformations. Gross examination of our mutants with *Wt1* deleted did not reveal any major malformations, but it is possible that some abnormalities might not be obvious until the pups are older. Mortality rates in infants with CDH remain high despite advances in surgical repair. Pulmonary complication is a major cause of CDH mortality. Herniated viscera compress the lung and lead to pulmonary hypoplasia. However, abnormal lung development can precede the herniation of abdominal content into the thoracic cavity, suggesting that it can be a primary rather than a secondary defect (Donahoe et al., 2016). Lung hypoplasia has been observed in the mouse model where *Wt1* is inactivated globally (Kreidberg et al., 1993). Relating to our model, the *Prx1-Cre* transgene has been shown to label some cells in the lungs (Merrell et al., 2015); however, the exact cell type is not clear. By contrast, *Wt1-*expressing cells have been shown to contribute to pulmonary endothelial and smooth muscle cells, bronchial musculature, tracheal and bronchial cartilage, and CD34^+^ fibroblast-like interstitial cells (Cano et al., 2013). Despite the compelling evidence suggesting that our model is also likely to have a lung phenotype, we cannot be sure without a detailed analysis.

Other components that are also crucial in diaphragm development are blood vessels and the nervous system (Merrell and Kardon, 2013), but these have not been analysed in our model. Furthermore, with advances in single-cell RNA-seq technique, analysis of diaphragm development using this approach will provide further insight into the cellular heterogeneity of the diaphragm, in addition to the pathways, regulatory mechanisms and possible interactions between different cell types during diaphragm development.

In summary, we illustrate the heterogeneous nature of the cell population that forms the PPFs, using several new mouse models. We show that the conditional deletion of *Wt1* in the non-muscle mesenchyme using *Prx1-Cre* results in diaphragmatic hernias, and we illustrate that the mesenchymal cells of the PPFs are capable of expanding towards the PHMP. Connective tissue fibroblasts are absent in the PPFs in which the non-muscle mesenchyme is deleted for *Wt1*, suggesting possible interactions between these two cell types. This absence of connective tissue fibroblasts could be a plausible explanation for the evident disruption to the migration; however, more work is required to delineate the exact underlying molecular mechanism.

## MATERIALS AND METHODS

### Mouse husbandry

Animals used in this study were housed at the animal facilities at the University of Edinburgh, with procedures performed under Personal and Project Home Office Licences. Targeted deletion of *Wt1* (*Prx1^Cre^*^/+^*;Wt1^loxp/loxp^*) was achieved by crossing homozygous mice (Martínez-Estrada et al., 2010) carrying a loxP-flanked *Wt1* allele into a mouse strain of Cre recombinase expression driven by the *Prx1* transgene (Logan et al., 2002). The *Wt1-GFP* mouse line (*Wt1^GFP^*^/+^) used in this study was made by Hosen et al. (2007). GFP is a knock-in at the first exon of *Wt1* and is expressed under the endogenous transcriptional regulatory elements of *Wt1*. The *Prx1-Cre* lineage-tracing model (*Prx1^Cre^*^/+^*;R26R^mTmG^*^/+^) was made by crossing male *Prx1-Cre* mice (Logan et al., 2002) with female *R26R^mTmG/mTmG^* double fluorescence reporter mice (Muzumdar et al., 2007). Cre expression, following *Prx1*-derived enhancer element, mediates a switch of Tomato to eGFP expression. The model to obtain mutant diaphragmatic cells that are expressing WT1 and have been conditionally deleted for *Wt1* by the *Prx1-Cre* (*Prx1^Cre^*^/+^*;W1^GFP/loxp^;R26R^tdRFP^*^/+^) was generated by crossing *Prx1^Cre^*^/+^*;Wt1^GFP^*^/+^*;R26R^tdRFP/tdRFP^* male with female *Wt1^loxp/loxp^* mice. To obtain counterpart control cells in the above model, male *Prx1^Cre^*^/+^*;Wt1^GFP^*^/+^*;R26R^tdRFP/tdRFP^* mice were crossed with wild-type females. The *R26R^tdRFP^*^/+^ line was generated as described by Luche et al. (2007).

### Tissue preparation

Samples were fixed in 4% paraformaldehyde (dissolved in PBS) at 4°C overnight, unless otherwise stated. The next day, samples were washed three times in PBS (10 min each) and stored in 70% ethanol before preparation for paraffin embedding. A Tissue-Tek VIP Jr. Vacuum Infiltration Processor was used for paraffin wax embedding. Paraffin sections were cut at a thickness of 5-6 μm using a microtome, mounted on SuperFrost Plus Microscope slides and dried at 50°C overnight.

### Immunofluorescence

Immunofluorescence staining was performed using a similar protocol to the one described previously (Chau et al., 2014). The primary antibodies used in this study were anti-WT1 (Abcam, ab89901, 1:1000), anti-Ki67 (Abcam, ab15580, 1:1000), anti-GFP (Abcam, ab5450, 1:1000), anti-PAX2 (Biolegend, 901001, 1:100), anti-MF20 (DSHB, Ab_2147781, 1:20), anti-VIM (Santa Cruz Biotechnology, sc-7557, 1:100), anti-CDH1 (BD, 610181, 1:100), anti-RALDH2 (Santa Cruz Biotechnology, sc-22591, 1:200), anti-GATA4 (Santa Cruz Biotechnology, sc-25310, 1:100), anti-TCF4 (Cell Signaling Technology, C48H11, 1:100) and anti-MYOD (Santa Cruz Biotechnology, sc-32758, 1:100). Alexa-Fluor 488- or 594-conjugated antibodies were used as secondary antibodies. Sections were stained with 4′,6-diamidino-2-phenylindole (DAPI) and mounted using VectorShield. A Zeiss Axioplan II microscope was used to view immunofluorescence and H&E-stained sections. Image capture was performed using the open source microscopy software: uManager.

### H&E staining

Slides were dewaxed in xylene and rehydrated in a series of ethanol washes, followed by washing in tap water and staining with Mayer's Haematoxylin. After washing in tap water, sections were differentiated in 1% HCl in 70% ethanol for a few seconds and washed with tap water. Slides were stained with saturated lithium chloride solution for a few seconds before washing in tap water. Slides were then stained with Eosin, rinsed in tap water, rehydrated and mounted using DPX mounting medium.

### Flow cytometry and FACS

Tissues of the thoracic region/upper abdominal region (with lung, heart and liver removed) were digested into a single-cell suspension using collagenase (1 mg/ml collagenase and 4 mg/ml bovine serum albumin, dissolved in PBS) for 30-60 min at 37°C with shaking. Collagenase activity was stopped by washing the cells in PBS containing 5% fetal calf serum. Cells were pelleted by centrifugation at 300 ***g*** for 5 min. Cells were filtered using a 40 µm cell strainer and subjected to FACS (BD FACSAria II System). RNA from the sorted cells was extracted using TRIzol reagent.

### Quantitative PCR

Complementary DNA was synthesised from RNA using a QuantiTect Whole Transcriptome kit (Qiagen), following the manufacturer's protocol. Quantitative PCR was performed using the Universal Probe Library system (Roche) using a LightCycler 480 II machine. The cycling conditions were pre-incubation (10 min, 95°C), amplification (95°C for 10 s, 60°C for 30 s and 72°C for 1 s) and cooling (40°C for 30 s). The amplification was repeated 50 times. Primer and probe information for each gene used is listed in Table S3.

## Supplementary Material

Supplementary information
