# Supplementary material for: Resolving the heterogeneity of diaphragmatic mesenchyme: a novel mouse model of congenital diaphragmatic hernia
Source: Dis Model Mech. 2021 Jan 26;14(1):dmm046797. doi: 10.1242/dmm.046797 (PMC7859704; doi:10.1242/dmm.046797)
Supplement: Supplementary information [file dmm-14-046797-s1.pdf]

Table S1. *The number of the ($Prx1^{Cre/+}$;$Wt1^{loxp/loxp}$) embryos obtained at each developmental stage, compared with the expected number.*

EMBRYO STAGE	TOTAL # EMBRYOS	# EXPECTED MUTANTS	# ACTUAL MUTANTS
E10.5	27	6.75 (25%)	6 (22%)
E11.5	18	4.5 (25%)	4 (22%)
E12.5	26	6.5 (25%)	6 (23%)
E14.5	9	2.25 (25%)	3 (33%)
E16.5	22	5.5 (25%)	8 (36%)
E18.5	6	1.5 (25%)	1 (17%)
E19.5	25	6.25 (25%)	4 (16%)
P0	24	6 (25%)	4 (16%)

Disease Models & Mechanisms • Supplementary information

Table S2. *Phenotype descriptions for the (Prx1$^{Cre/+}$;Wt1$^{loxp/GFP}$) embryos and pups at different developmental stages*

EMBRYO STAGE	TOTAL # MUTANTS	GENDER/DIAPHRAGM PHENOTYPES
E14.5	3	Hole left side, dorsal, with liver herniation.
		Hole left side, ventral, with liver herniation.
		Hole in right side dorsal, possible liver herniation.
E16.5	8	Female. Bilateral holes, dorsal, with liver herniation.
		Male. Hole left side, dorsal, with liver herniation.
		Female. No obvious hole/hernia – possible thinning of diaphragm on left side. NB: Embryo sectioned in the coronal plane.
		Male. No obvious hole/hernia. Possible thinning at edges.
		Male. No obvious hole/hernia.
		Female. Bilateral holes, dorsal, with liver herniation.
		Female. No obvious hole/hernia.
		Male. No obvious hole/hernia.
E18.5	1	Bilateral thinning of the diaphragm, dorsal. No obvious hole/herniation.
E19.5	4	Bilateral holes, dorsal, with liver herniation.
		Small bilateral holes/thinning of the diaphragm, dorsal.
		Hole left side, dorsal.
		Hole left side, dorsal.
P0	4	Male. Large hole left side, dorsal, with stomach and liver herniation.
		Male. Large hole left side, dorsal, with stomach and liver herniation.
		Female. Large hole left side, dorsal, with stomach and liver herniation.
		Female. Large hole left side, dorsal, with stomach and liver herniation.

Table S3: Primer sequences and UPL probes

Gene	Primer (5' to 3')	Probe
Snai1	cttgtgtctgcacgacctgt, aggagaatggcttctcacca	71
Snai2	cattgccttgtgtctgcaag, cagtgagggcaagagaaagg	71
18S rRNA	cgattggatggtttagtgagg, agttcgaccgtcttctcagc	81

Supplementary Figure 1

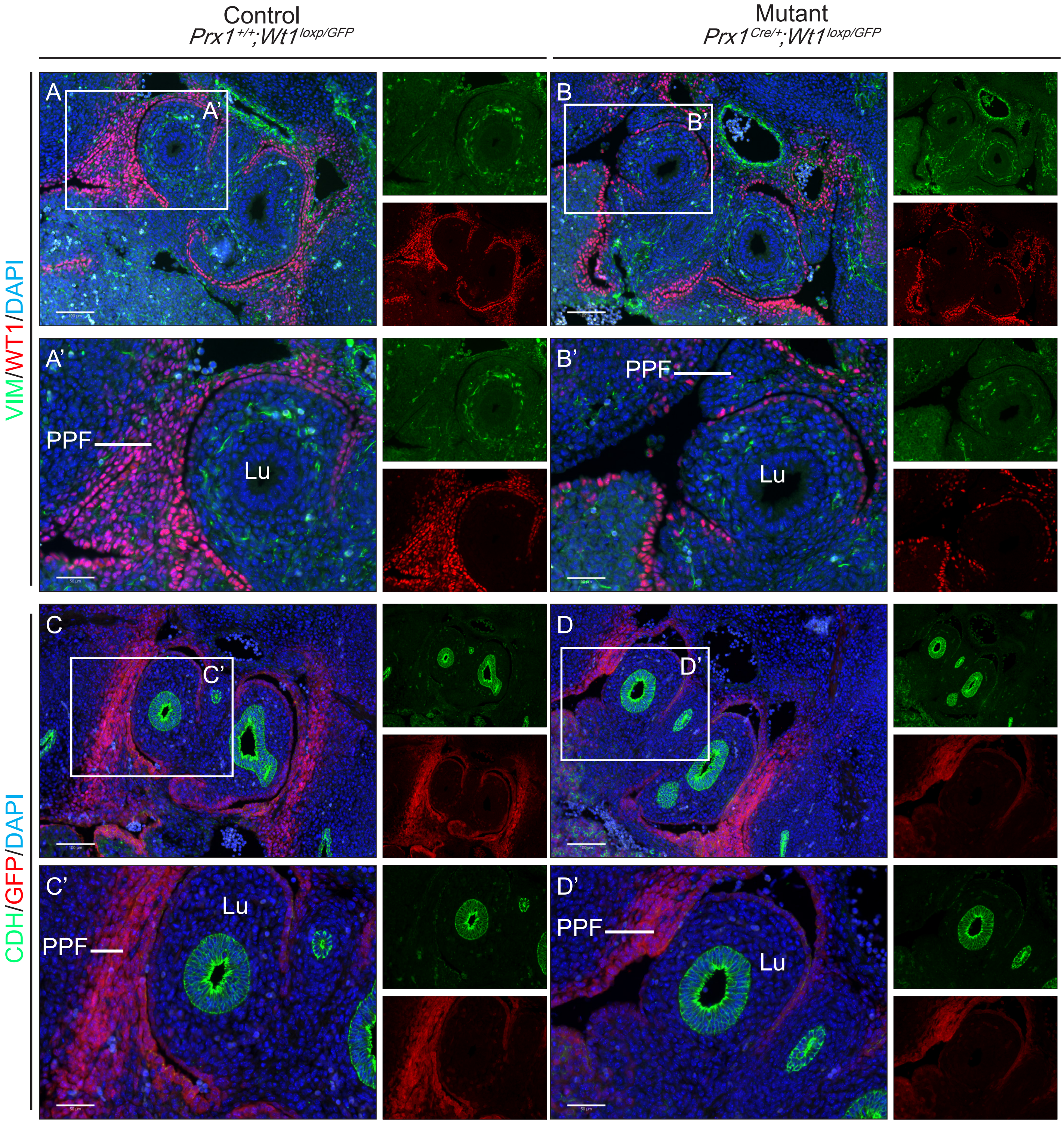

Disease Models & Mechanisms • Supplementary information

Figure S1. ***Wt1*** **deletion in the PPFs does not affect VIM or CDH1 expression in** ***Prx1**$^{Cre/+}$**;Wt1**$^{GFP/loxp}$* **embryos**. E11.5 embryos were sectioned and processed for immunofluorescence. (**A, A', B, B'**) Sections were stained with an anti-VIM antibody (vimentin, indicated in green) and an anti-WT1 antibody (indicated in red). WT1 expression was lost from the PPFs in mutant embryos but no difference in VIM expression was observed. Boxed areas in **A**,**B** are magnified in **A'**,**B'**. (n=3 animals for controls and n=4 animals for mutants). (**C, C', D, D'**) Sections were stained with an anti-CDH1 antibody (indicated in green) and an anti-GFP antibody (indicated in red). No difference in CDH1 (E-cadherin) expression was observed between control and mutant embryos. Boxed areas in **C,D** are magnified in **C'**,**D'**. (Abbreviations: Lu: Lungs, PPF: pleuroperitoneal fold. Scale bar: 100 μm in A-D, 50 μm in A'-D'). (n=3 animals for controls and 4 for mutants).

Supplementary Figure 2

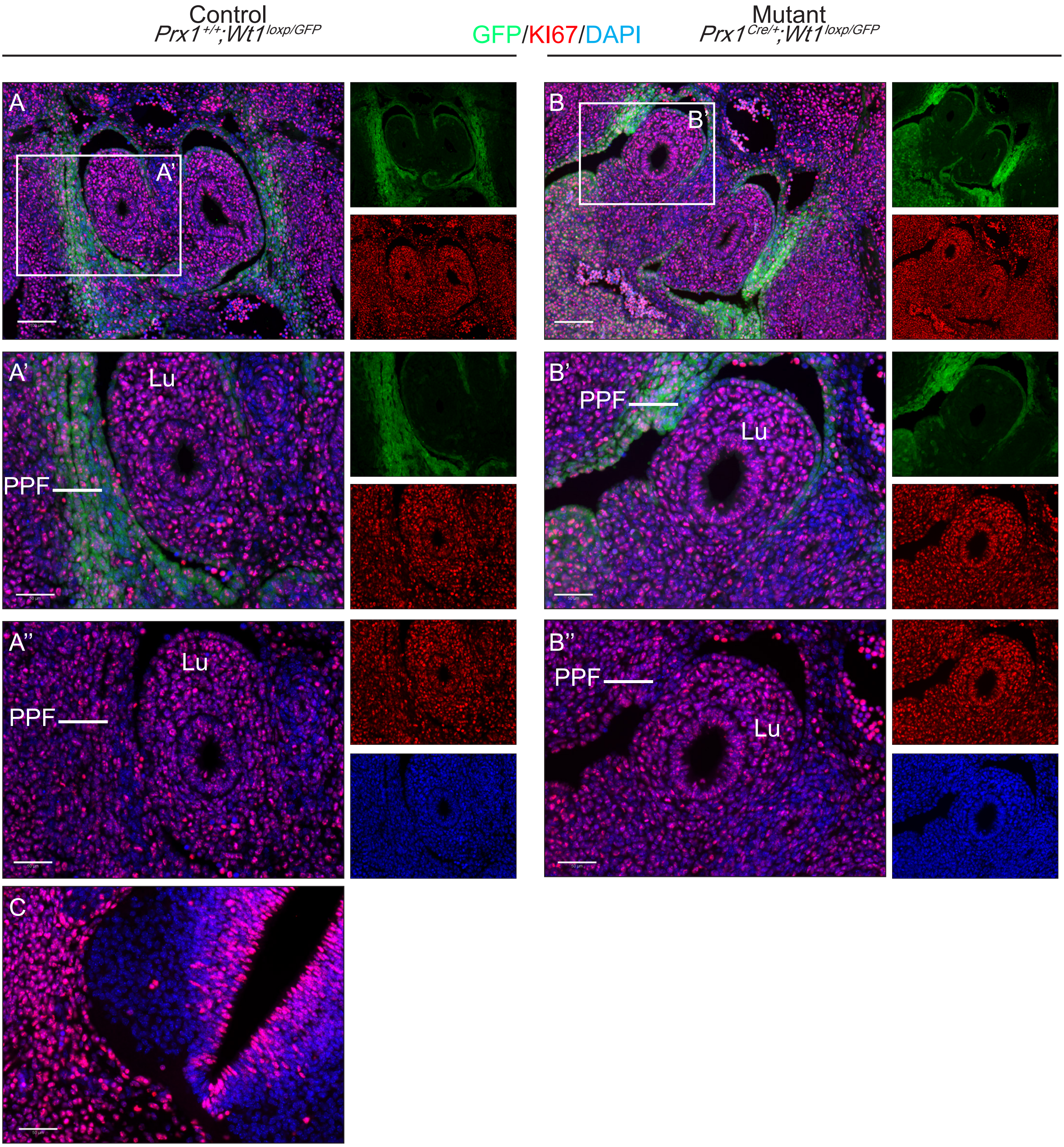

Disease Models & Mechanisms • Supplementary information

Figure S2. *Wt1* deletion in the PPFs does not affect cell proliferation in *Prx1$^{Cre/+}$;Wt1$^{GFP/loxp}$* embryos. E11.5 embryos were sectioned and stained with an anti-KI67 antibody (indicated in red) and an anti-GFP antibody (indicated in green). Cell nuclei are stained with DAPI (blue). Sections from control embryos are shown in (**A**, **A'**, **A''**) and mutant shown in (**B**, **B'**, **B''**). There is no difference between the number of KI67 positive cells between control and mutant embryos. (**A'** and **B'**) are magnified images of boxed areas shown in **A** and **B**. (**A''** and **B''**) show only KI67 staining (red) and DAPI (blue) for easier visualization. (**C**) shows a region containing neural tube to illustrate the specificity of the KI67 antibody. The asterisk highlights cells in neural tube that did not stain positive for KI67. (Abbreviation: Lu: Lungs, PPF: pleuroperitoneal fold. Scale bar: 100 μm in A,B; 50 μm in the rest of panels). (n=3 animals for each genotype).

Supplementary Figure 3

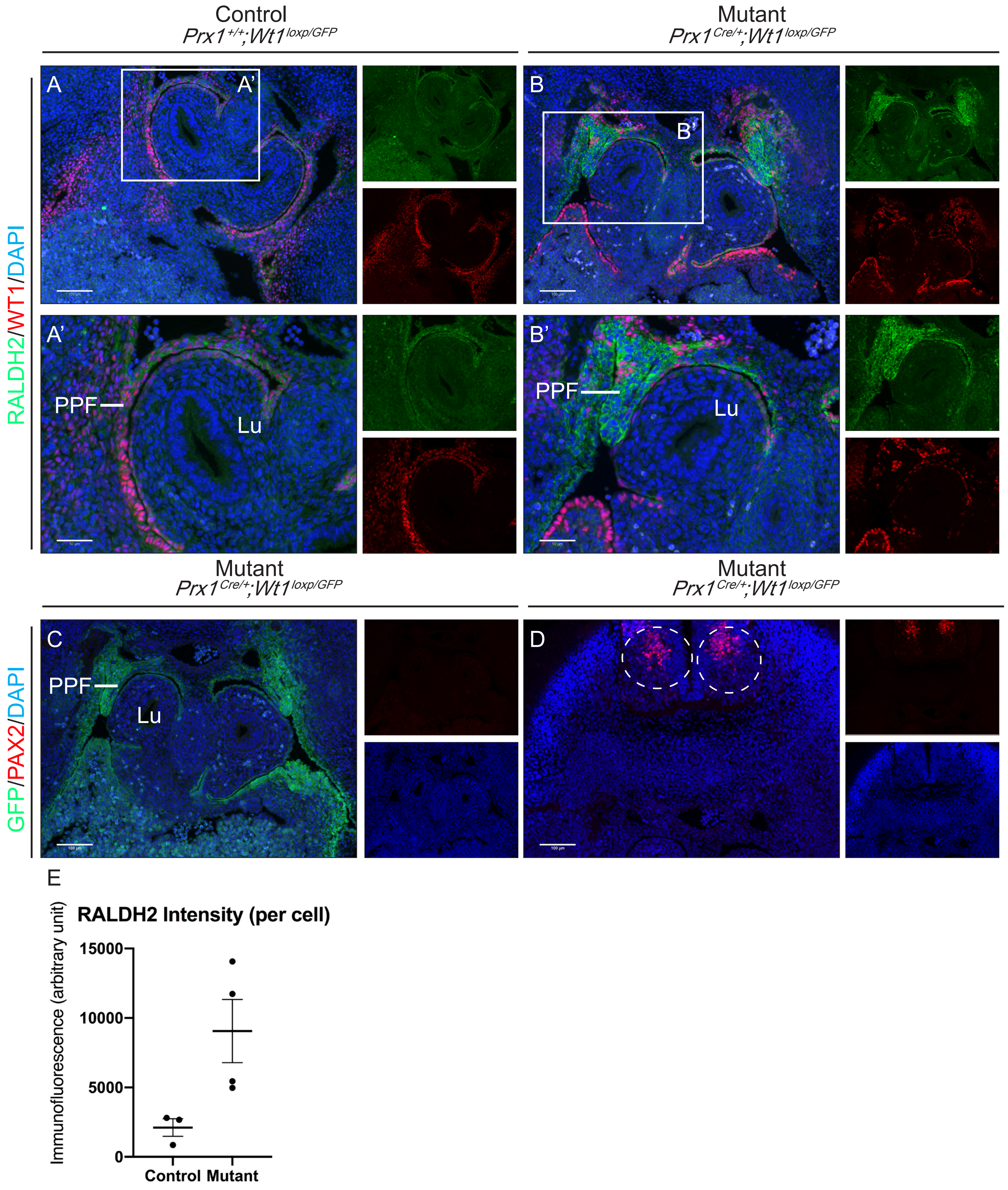

Disease Models & Mechanisms • Supplementary information

Figure S3. The PPFs deleted for *Wt1* exhibited increased RALDH2 and no alteration in PAX2 expression in *Prx1*$^{Cre/+}$*;Wt1*$^{GFP/loxp}$ embryos compared to littermate controls. E11.5 embryos were sectioned and processed for immunofluorescence. (**A, A', B**, **B'**) Sections were stained with an anti-RALDH2 antibody (indicated in green), an anti-WT1 antibody (indicated in red), and DAPI (blue). WT1 expression was lost from the PPFs in the mutant (**B, B'**), and RALDH2 expression was increased. **A'** and **B'** are magnified boxed areas indicated in **A** and **B**. (At E11.5, n=2 animals for controls and n=5 animals for mutants were analysed. At E12.5, n=3 animals for each genotype was analysed. (**C, D**) Sections were stained with an anti-PAX2 antibody (indicated in red), an anti-GFP antibody (green), and DAPI (blue). The lack of PAX2 expression in the PPFs suggests that they are not intermediate mesoderm (**C**). The neural tube is shown in (**D**) to illustrate that the anti-PAX2 antibody is working. (Abbreviations: Lu: Lungs, PPF: pleuroperitoneal fold; Scale bar: 50 μm in A' and B', 100 μm in the rest of panels). (n=2 animals for each genotype). (**E**) Quantification of RALDH2 immunofluorescence intensity (normalised by cell number, n=3 animals for control and n=4 animals for mutant).

Supplementary Figure 4

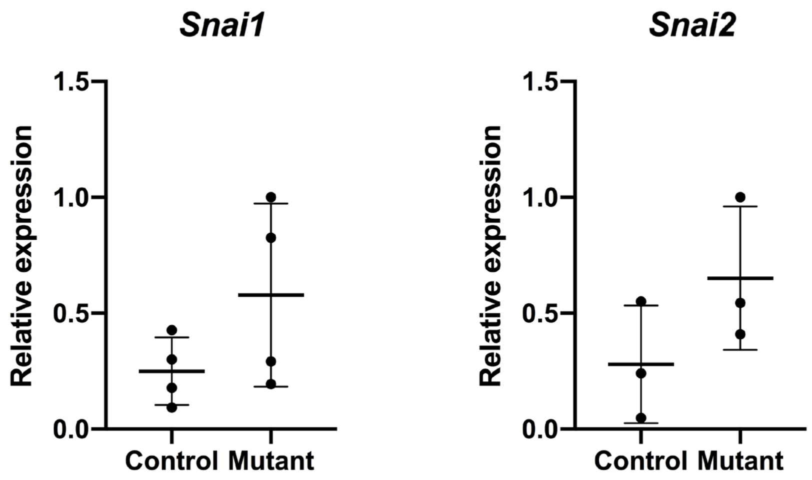

Figure S4. No alterations to mesenchymal markers in PPFs mesenchymal cells deleted for *Wt1 compared to littermate controls.* Mesenchymal cells in the E11.5 PPFs that are deleted for *Wt1* by *Prx1-Cre* in the model of (*Prx1*$^{Cre/+}$;*Wt1*$^{GFP/loxp}$;*R26R*^{tdRFP}/+) were FACS sorted for RNA purification and cDNA synthesis. Levels of Snail (*Snai1)* and Slug (*Snai2)* expression were measured by QPCR analysis. Control samples are GFP+RFP + cells FACS sorted from (*Prx1*$^{Cre/+}$;*Wt1*$^{GFP/+}$; *R26R*^{tdRFP}/+) where one copy of the *Wt1* allele contains a GFP knockin and the other allele is a wild type. Mutant samples are GFP+RFP+ cells FACS sorted from (*Prx1*$^{Cre/+}$;*Wt1*$^{GFP/loxp}$;*R26R*^{tdRFP}/+) embryos, where one copy of *Wt1* allele contains a GFP knockin while the second allele contains loxp sites flanking exon 1. For *Snai1* QPCR, n=4 animals for each genotype were analysed. Error bars indicate s.d.; For Snai2 QPCR, n=3 animals for each genotype were analysed. Error bars indicate s.d.

Supplementary Figure 5

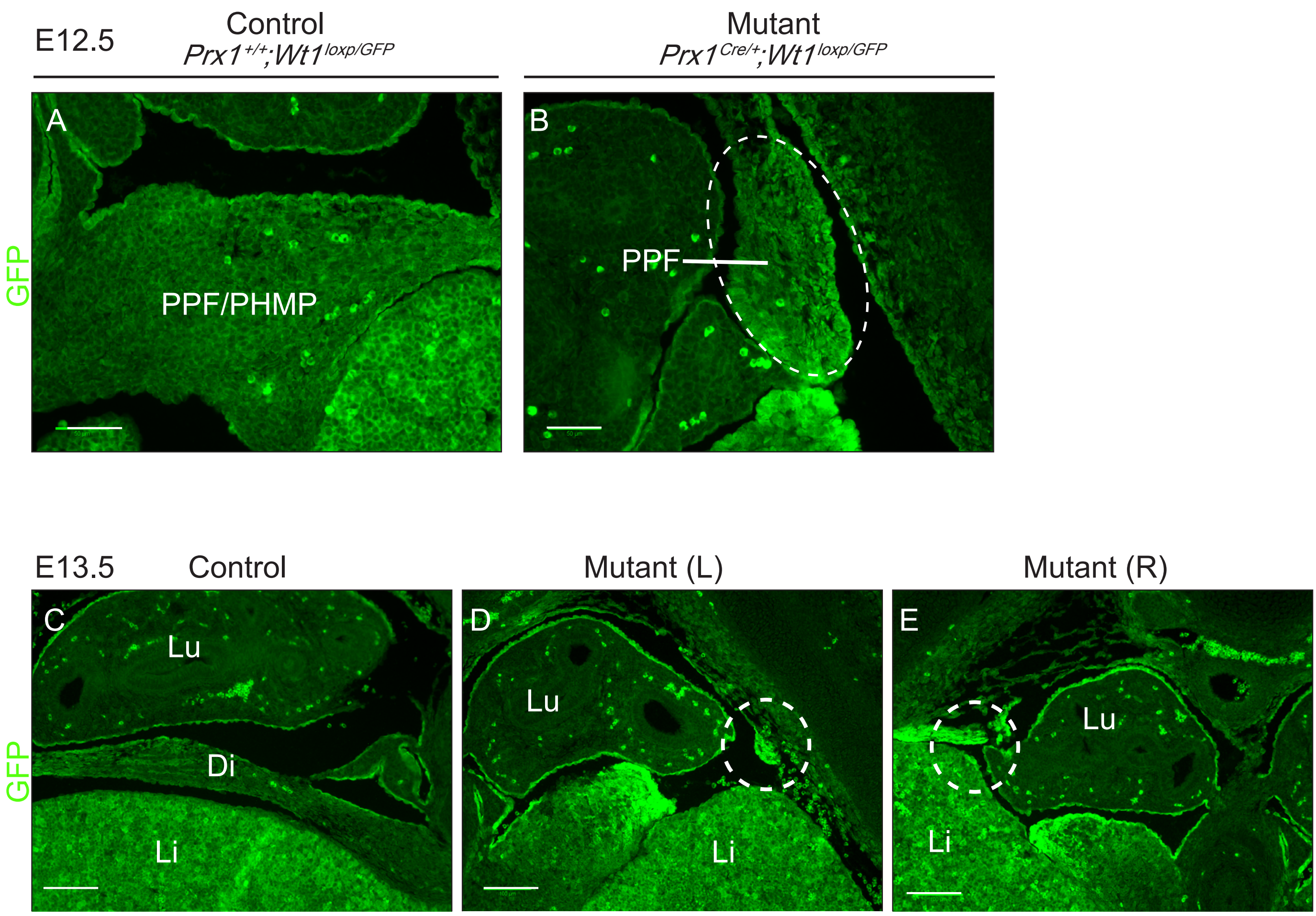

Figure S5. Corresponding single-channel images of Figure 5. Representative images of E12.5 and E13.5 embryos were sectioned and stained with an anti-TCF4 antibody (red), anti-GFP antibody (green), and DAPI (blue). Images representing the green channel (anti-GFP antibody) are shown. *(Abbreviations: Lu, lungs; Li, liver; Di, diaphragm; PPF, pleuroperitoneal fold; PHMP, posthepatic mesenchymal plate)*. (Scale bar = 50 μm in A,B; 100 μm in C-E). (n=3 animals for each genotype).

Disease Models & Mechanisms • Supplementary information

Supplementary Figure 6

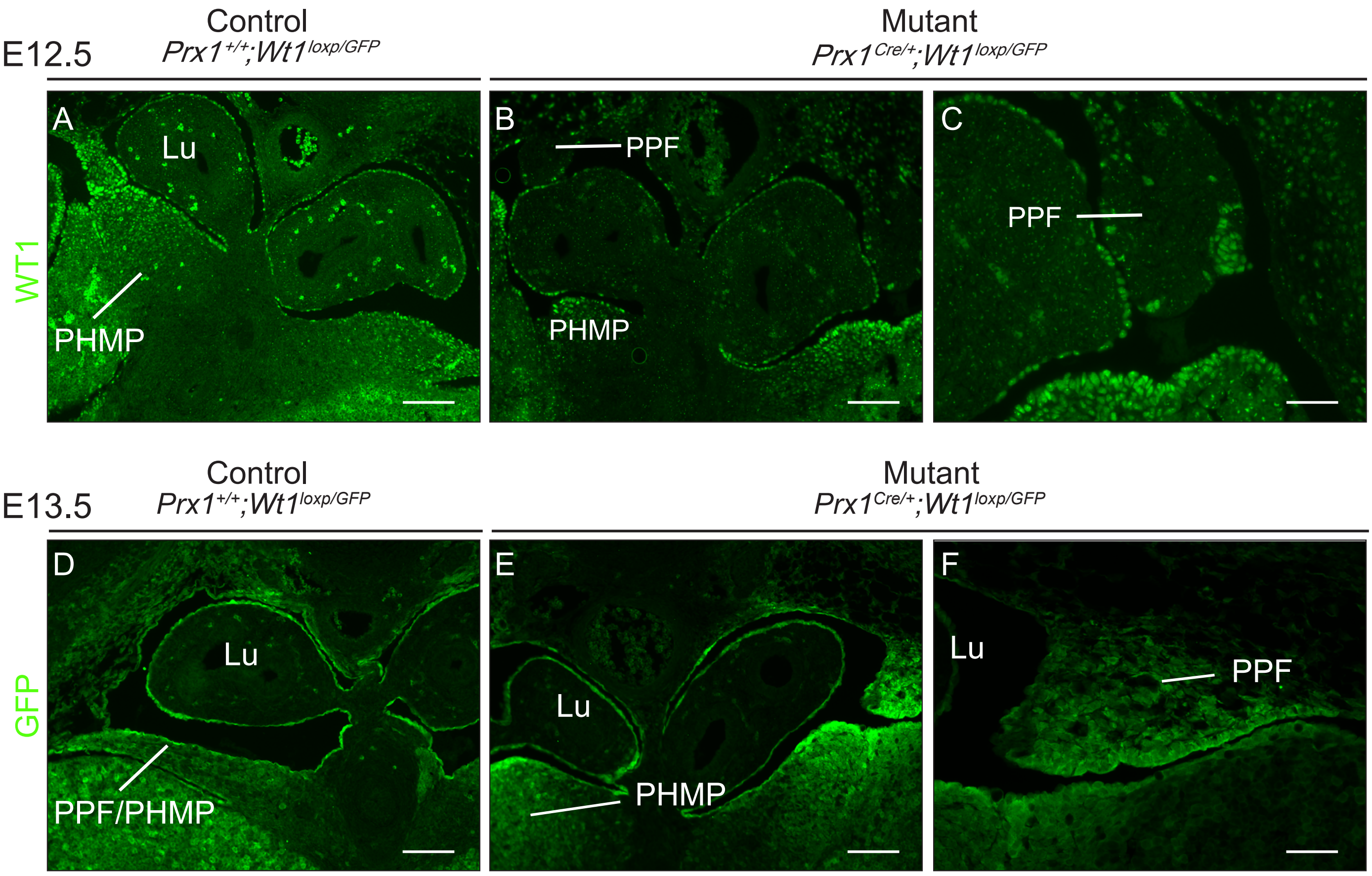

Figure S6. Corresponding single-channel images of Figure 6. In **(A-C)**, representative images of E12.5 embryos were sectioned and stained with an anti-GATA4 antibody (red), anti-WT1 antibody (green), and DAPI (blue). Single-channel images representing WT1 expression (green) are shown. In **(D-F)**, representative images of E13.5 embryos were sectioned and stained with an anti-GATA4 antibody (red), anti-GFP antibody (green), and DAPI (blue). Images representing GFP expression (green) are shown. *(Abbreviations: Lu, lungs; PPF, pleuroperitoneal fold; PHMP, posthepatic mesenchymal plate)*. (Scale bar = 50 μm in A, A', B, B'; 50 μm C, C', D, D', E, E'). (Scale bar: 50 μm in A,B,D, and E; the rest is 100 μm).

Disease Models & Mechanisms • Supplementary information

Supplementary Figure 7

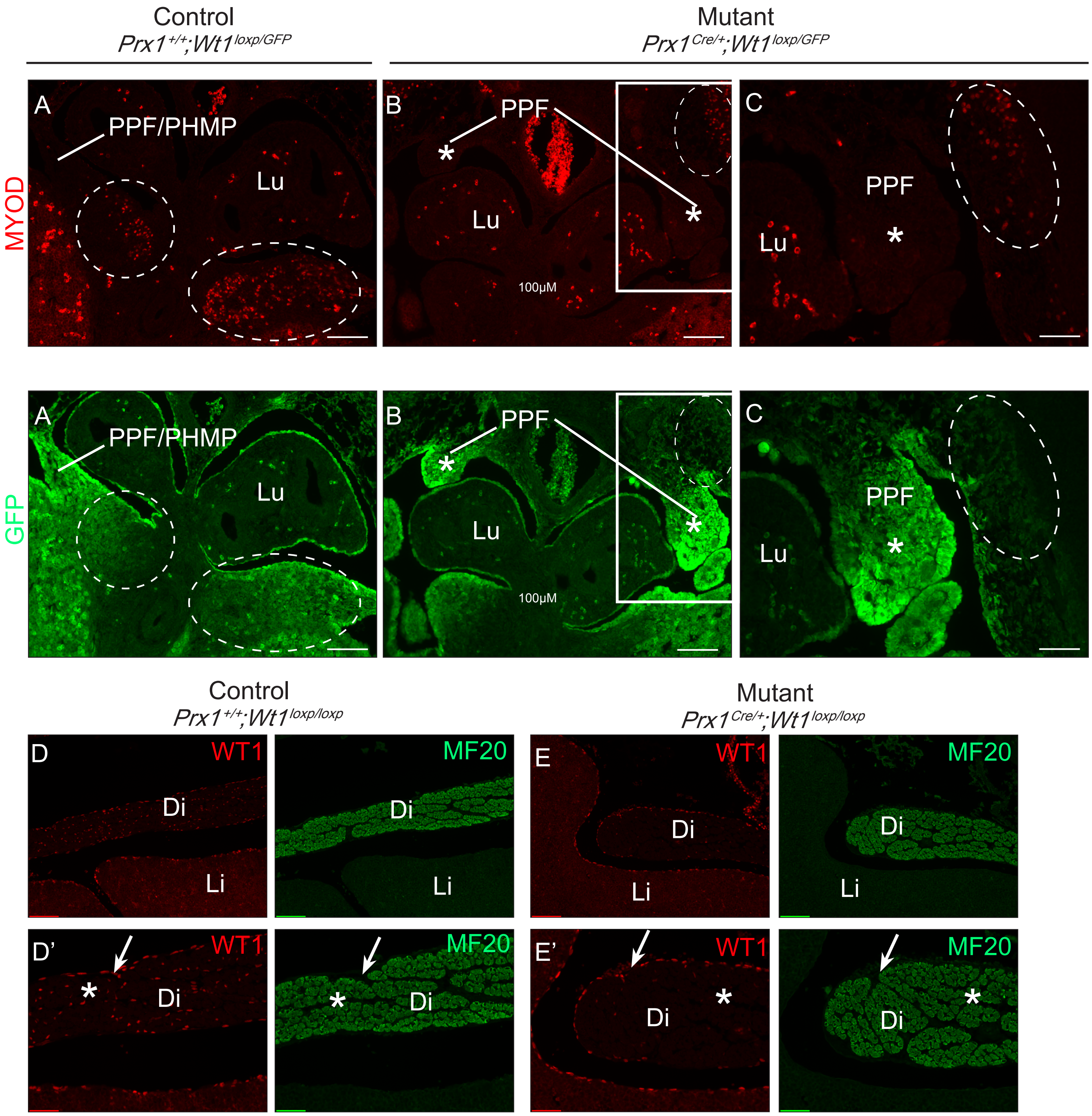

Disease Models & Mechanisms • Supplementary information

Figure S7. Corresponding single-channel images of Figure 7. In **A-C**, representative images of E12.5 embryos were sectioned and stained with an anti-MYOD antibody (red), an anti-GFP antibody (green), and DAPI (blue). Single-channel images representing MYOD (red) and GFP (green) expression are shown. (**D, D', E, E'**) Sagittal plan sectioned E16.5 embryos are stained with an anti-MF20 (green) and an anti-WT1 antibody (red). Single-channel images are shown. *(Abbreviations: Lu, lungs; PPF, pleuroperitoneal fold; PHMP, posthepatic mesenchymal plate, Li: liver, Di: diaphragm)*. (Scale bar = 100 μm in A, B, D, and E; 50 μm in C, D', and E'). Together, n=3 animals for controls and n= 6 animals for mutants were analysed for MF20 staining.
